# CDC Laboratory Recommendations for Syphilis Testing, United States, 2024

**DOI:** 10.15585/mmwr.rr7301a1

**Published:** 2024-02-08

**Authors:** John R. Papp, Ina U. Park, Yetunde Fakile, Lara Pereira, Allan Pillay, Gail A. Bolan

**Affiliations:** ^1^Division of STD Prevention, National Center for HIV, Viral Hepatitis, STD, and TB Prevention, CDC, Atlanta, Georgia; ^2^University of California San Francisco, San Francisco, California; ^3^The Task Force for Global Health, Decatur, Georgia

## Abstract

*This report provides new CDC recommendations for tests that can support a diagnosis of syphilis, including serologic testing and methods for the identification of the causative agent* Treponema pallidum*. These comprehensive recommendations are the first published by CDC on laboratory testing for syphilis, which has traditionally been based on serologic algorithms to detect a humoral immune response to* T. pallidum*. These tests can be divided into nontreponemal and treponemal tests depending on whether they detect antibodies that are broadly reactive to lipoidal antigens shared by both host and* T. pallidum *or antibodies specific to* T. pallidum*, respectively. Both types of tests must be used in conjunction to help distinguish between an untreated infection or a past infection that has been successfully treated. Newer serologic tests allow for laboratory automation but must be used in an algorithm, which also can involve older manual serologic tests. Direct detection of* T. pallidum *continues to evolve from microscopic examination of material from lesions for visualization of* T. pallidum *to molecular detection of the organism. Limited point-of-care tests for syphilis are available in the United States; increased availability of point-of-care tests that are sensitive and specific could facilitate expansion of screening programs and reduce the time from test result to treatment. These recommendations are intended for use by clinical laboratory directors, laboratory staff, clinicians, and disease control personnel who must choose among the multiple available testing methods, establish standard operating procedures for collecting and processing specimens, interpret test results for laboratory reporting, and counsel and treat patients.* *Future revisions to these recommendations will be based on new research or technologic advancements for syphilis clinical laboratory science.*

## Introduction

### Background

*Treponema pallidum* subsp. *pallidum,* primarily transmitted through sexual contact, is among four pathogenic species in the genus *Treponema,* which is in the family *Treponemataceae* (*1*). The other three pathogenic *Treponema* species cause skin diseases mostly transmitted by direct skin-to-skin contact. Yaws is caused by *T. pallidum* subsp. *pertenue* and is found in tropical areas in Africa, Asia, and Latin America (*2*). *Treponema carateum* infection results in pinta which, although rare, is found in tropical areas of Latin America (*3*). Endemic syphilis or bejel, caused by *T. pallidum* subsp. *endemicum*, occurs mostly in children and is mainly found in the eastern Mediterranean, West Africa, and Cuba (*4*,*5*). However, phylogenic analysis of lesion specimens from certain patients outside of areas where bejel is endemic who had received a diagnosis of syphilis revealed that *T. pallidum* subsp. *endemicum* might be sexually transmitted. These patients have a clinical course similar to syphilis (*5*–*8*). For this report, *T. pallidum* subsp. *pallidum* will be abbreviated to *T. pallidum* unless further distinction between the subspecies is necessary.

*T. pallidum* causes a systemic infection and might lead to serious sequalae in multiple organ systems, including the central nervous system (CNS) and the ocular and otic systems. Vertical transmission can cause congenital syphilis, which might result in spontaneous abortions, miscarriages, or stillbirths; infants with congenital syphilis can have clinical signs of infection at birth or months to years after birth. Clinical features in adults progress through different stages beginning with primary syphilis, which often appears about 3 weeks after exposure, with an incubation period of 10–90 days (*9*). Primary syphilis is characterized by single or multiple ulcerative-like lesions (chancres) that often are painless and therefore might be unnoticed when they occur inside the mouth, vagina, or rectum. Chancres can persist for 2–6 weeks before healing spontaneously. Secondary syphilis typically begins 2–24 weeks after most primary lesions heal and is commonly characterized by a mucocutaneous rash appearing on the trunk, palms, and soles; mucous patches in the mouth or condylomata lata on the genitals or rectum occur in approximately one fourth of patients. Primary and secondary syphilis symptoms can occur concurrently, which is more likely in persons with HIV infection. Moist primary and secondary syphilis lesions contain infectious *T. pallidum* that can be transmitted through sexual contact to susceptible persons. Secondary clinical manifestations also can consist of lymphadenopathy, alopecia, and occasionally neurologic and ocular manifestations. Signs and symptoms of secondary syphilis typically resolve in approximately 3 months, with a range of 1–12 months (*10*,*11*) but can periodically recur for the first several years of infection in ≤25% of untreated persons (*12*).

The interval between primary to secondary and secondary to tertiary syphilis is known as latency when no symptoms or signs of syphilis are present. The interval from secondary to tertiary syphilis can last for years or decades before symptoms appear. In up to two thirds of patients, the disease can remain latent for life and never progress to tertiary syphilis (*13*–*15*). Latent asymptomatic syphilis is divided into three categories: early latent infections thought to have been acquired within the past year; late latent infections thought to be longer than 1 year duration; and latent syphilis of unknown duration where the timing of acquisition cannot be determined based on available clinical, historical, or laboratory data. Clinical signs of tertiary syphilis, a rare condition, include cardiovascular syphilis, with aneurysms or stenosis resulting from multiplication of treponemal spirochetes in the thoracic aorta or coronary arteries; syphilitic gummas, with soft granulomatous growths that can cause tissue destruction in any organ system including bones and cartilage; and neurosyphilis, with late neurologic manifestations including tabes dorsalis and general paresis. Neurosyphilis can occur during any stage of syphilis and can be asymptomatic or symptomatic during any stage of infection.

### Rationale for New CDC Recommendations

Syphilis, a nationally notifiable disease with approximately 176,000 cases in the United States reported to the CDC in 2021 (*16*) and approximately 6 million new cases occurring worldwide (*17*), is caused by *T. pallidum*. A syphilis epidemic is occurring in the United States, with sustained increases in primary and secondary syphilis from 5,979 cases reported in 2000 to 133,945 cases reported in 2020, a 2,140% increase (*16*,*18*). The epidemic is characterized by health disparities, particularly among sexual and gender minority populations, intersections with the HIV and substance use epidemics, and increased morbidity and mortality attributable to congenital syphilis infections (*16*).

Laboratories have a critical role in the public health response to the syphilis epidemic. The responsibility of the laboratory is to test specimens and report results in a timely manner, allowing clinicians to efficiently make clinical diagnoses for patient management. Public health reporting by laboratories also allows local health departments and CDC to conduct surveillance and monitor disease trends. This report details CDC’s new recommendations for syphilis testing, including laboratory-based tests, point-of-care (POC) tests, processing of samples, and reporting of test results to aid laboratorians and clinicians in the diagnosis of syphilis. Future revisions to these recommendations will be based on new research or technologic advancements for syphilis clinical laboratory science.

## Methods

These recommendations were developed by CDC staff members on the basis of evidence published in peer-reviewed scientific journals. Data available in Food and Drug Administration (FDA)-cleared syphilis diagnostic test inserts were reviewed and assessed for consistency with published findings. In 2017, the Association of Public Health Laboratories (APHL) assisted with the literature review through an independent work group formed to evaluate the scientific literature for CDC to consider in the development of evidence-based recommendations for syphilis testing in the United States. APHL work group members were selected based on expertise in the field of syphilis and represented public health and commercial laboratory directors, public- and private-sector providers, and academic researchers. The work group leads were experienced in conducting systematic reviews of the literature. Potential conflicts of interest were disclosed to APHL and are listed at the end of the work group (Supplementary Appendix 1, https://stacks.cdc.gov/view/cdc/138288). APHL staff members reviewed potential conflicts and concluded that no work group members had a financial interest or ongoing relationships that might bias the literature review and subsequent discussions. The APHL work group did not rank the evidence and did not make any recommendations based on the scientific literature review. CDC staff members involved in ranking the evidence and drafting recommendations based on the scientific literature certified that they did not have a perceived or actual competing interest with respect to this activity.

CDC identified key questions regarding syphilis testing in the United States that should be addressed during the literature review process and shared these questions with the APHL work group members in March 2017. Work group members were assigned key questions to review (Supplementary Appendix 2, https://stacks.cdc.gov/view/cdc/138288) and, with the assistance of CDC and APHL staff members, conducted an extensive literature search on Medline, Embase, Scopus, Cochrane Library, and CINAHL; combinations of search terms for each key question were used to search for literature published during January 1–June 30, 2017 (Supplementary Appendix 2, https://stacks.cdc.gov/view/cdc/138288). The wide time interval was necessary because certain tests have been used for almost a century. In November 2017, work group members presented their reviews to CDC and APHL staff members. Key questions and pertinent publications were reviewed for strengths, weaknesses, and relevance and were discussed by individual work group members. The discussions were informal and not designed to reach consensus; no formal rating system was used. Background papers summarizing the evidence reviewed were peer reviewed and published in July 2020 (*19*–*23*). Subsequently, CDC staff members used the same search criteria and evidence review ranking methods described previously to identify articles published through September 1, 2022.

After the November 2017 meeting, the APHL work group was disbanded. CDC staff members reviewed the scientific evidence and ranked the evidence as high, medium, or low on the basis of each study’s strengths and weaknesses as outlined by the U.S. Preventive Services Task Force Ratings (https://www.uspreventiveservicestaskforce.org/uspstf/us-preventive-services-task-force-ratings). Studies were rated A if they were high quality using clinically characterized specimens, were stratified by stage, had larger sample size, were prospective, or were well-done cross-sectional or retrospective studies. B-rated studies were good to moderate quality with large sample sizes, were clinically characterized but not stratified by stage, or were characterized but unclear exactly how it was done with mild methodological issues. C-rated studies were fair quality and included those with small sample sizes, moderate methodological issues, used a single laboratory test as gold standard, or were descriptive. D-rated studies were poor quality and included studies with major methodologic issues or small sample sizes. Case reports or small case studies were rated as I. Studies that were not relevant to the key question were assigned as NR and not further rated. The recommendations were based on high-ranking scientific evidence from A- and B-ranked studies that would result in a net benefit for the diagnosis of syphilis and ultimately patient care (Supplementary Tables 1, 2, 3, 4, 5, 6, and 7, https://stacks.cdc.gov/view/cdc/138288). CDC staff members considered harms and benefits to patients when formulating these recommendations so that studies with misleading or poor data that might lead to a net harm for patient care because of inaccurate laboratory testing were not included. Other factors (e.g., cost-benefit) also were considered and included in this report.

Draft recommendations were peer reviewed as defined by the Office of Management and Budget for influential scientific information (https://wcms-wp.cdc.gov/os/quality/support/peer-review.htm). In February 2022, draft recommendations were peer reviewed by four experts in the field of syphilis who were not U.S Federal employees, were not funded by CDC for syphilis research, and were not involved in the development of these recommendations (Supplementary Appendix 3, https://stacks.cdc.gov/view/cdc/138288). Comments submitted during the external peer review were addressed, and the document was available for a 60-day public comment period beginning April 5, 2023. Draft recommendations were reviewed by subject matter experts and stakeholders, including APHL, the American Society for Microbiology, the Centers for Medicare & Medicaid Services (CMS), and FDA. After the public comment and stakeholder review, CDC considered all comments in the development of final testing recommendations for syphilis.

## Updating Syphilis Serologic Laboratory Terminology

Syphilis serologic tests were developed at the beginning of the 20th century and used by medical personnel to diagnose syphilis. The first test, known as the Wassermann test, was a complement fixation test that used liver extracts, initially from fetuses and subsequently from the heart tissue of patients with syphilis (*24*). The assay was further standardized to improve reproducibility by laboratories after the publication of a method to isolate cardiolipin and lecithin (phosphorylcholine) from beef heart and combine them with cholesterol as the antigens for these tests (*25*). Subsequent tests involving immobilization of *T. pallidum*, agglutination, or flocculation were based on the same principle of detecting serum that reacted to *T. pallidum* (*T. pallidum* immobilization [TPI] test) or to antigens found in the membranes of *T. pallidum* (cardiolipin [diphosphatidylglycerol], phosphorylcholine, and cholesterol) used in the rapid plasma reagin (RPR) and Venereal Disease Research Laboratory (VDRL) tests. In 1954, the World Health Organization convened an expert committee on treponematoses and made recommendations regarding antigen preparation, standardization of tests, and terminology (*26*). The terminology was based on the understanding of the contemporaneous scientific findings and became the basis for which to describe the serologic testing concepts for syphilis that are still used today (*27*). Over time, the use of the terms nontreponemal tests, treponemal tests, and nonspecific antibodies should be revisited and updated to be consistent with the scientific evidence related to the immunobiology of *T. pallidum*.

### Immunobiology

*T. pallidum* are obligate microaerophilic spirochete bacteria with a flexuous, flat-wave morphology that range from 5 to 20 *μ*m in length and 0.1 to 0.4 *μ*m in diameter (*28*). The protoplasm is enclosed by a cell wall composed of a cytoplasmic membrane, a thin peptidoglycan layer, and a simple lipid bilayer outer membrane (*29*,*30*). The bacterial structure is similar to other gram-negative bacteria (e.g., a periplasmic space separates the cytoplasmic and outer membranes). However, in contrast to most other gram-negative bacteria, the outer membrane of *T. pallidum* is extremely fragile, lacks a lipopolysaccharide outer layer, has the peptidoglycan layer above the cytoplasmic membrane rather than beneath the outer membrane, and has approximately a 100-fold lower density of proteins that span the membrane (*2*,*31*–*36*). The organism exhibits corkscrew-like motility, rotating around its longitudinal axis that is provided by endoflagella located in the periplasmic space and wrapped around the cell body (*37*–*39*). The relatively few integral membrane proteins, exposed lipoproteins, and phospholipids likely make up the bacterial surface and contribute to its relative lack of surface antigenicity (*30*,*40*).

After entry through the mucosa or microabrasions in the skin, *T. pallidum* replicates locally and quickly spreads throughout the body, including the CNS, through the cardiovascular and lymphatic systems (*41*). The dearth of pathogen-associated molecular patterns on the cell surface of *T. pallidum* contributes to the inability of the innate immune system to clear the organism during primary infection and subsequent dissemination (*42*). Activation of the innate immune system might be downregulated by a treponemal phospholipid found in the outer membrane (*43*). However, dendritic cells phagocytize *T. pallidum* early during infection, and most migrate to draining lymph nodes where they present processed treponemal antigens (mostly protein antigens) to B- and T-cells to initiate adaptive immune responses (*44*).

Antigens that are processed and presented by phagocytic cells during *T. pallidum* infection are either unique to the organism or common to the organism, host cells, or both. Cardiolipin, a diphosphatidylglycerol, is an integral mitochondrial cell membrane phospholipid required for proper mitochondrial function (*45*). B1 cells, a subset of B-cells, secrete antibodies of low to moderate affinity in the absence of activation by previous infection (*46*). The B1-secreted antibodies are referred to as natural antibodies, and they can bind to cardiolipin and other phospholipids (e.g., cholesterol and phosphatidylcholine). However, other infections or conditions, in addition to syphilis and autoimmune diseases, can cause a transient increase in natural antibodies against cardiolipin (*47*). The cytoplasmic membrane of *T. pallidum* contains cardiolipin and other phospholipids that can contribute to immune stimulation during infection (*48*,*49*). Cholesterol and phosphatidylcholine are host phospholipids that are also constituent macromolecules in the *T. pallidum* cytoplasmic membrane (*48*). Phosphorylcholine can be a target for protective immunity, as demonstrated by the bactericidal effect of a monoclonal antibody binding to this antigen on the surface of *T. pallidum* (*50*). Antibodies to both cholesterol and phosphatidylcholine are elevated during certain stages of infection with *T. pallidum* (*51*) and are detected by RPR and VDRL tests.

### Syphilis Serologic Laboratory Testing Terminology

#### Nontreponemal Test

Antibodies that reacted to the lipoidal antigens used in the Wassermann and subsequent agglutination or flocculation tests were either an indication of a concomitant *T. pallidum* infection or another condition related to host tissue damage and release of lipoidal antigens. The term nontreponemal test was first used in the literature in 1960 to differentiate tests based on antigens specific to *T. pallidum* (TPI, fluorescent treponemal antibody-absorption [FTA-ABS], microhemaggluntination assay for antibodies to *T. pallidum* [MHA-TP], *T. pallidum* hemagglutination assay [TPHA], and *T. pallidum* particle agglutination [TPPA]) from tests based on antigens (i.e., cardiolipin, phosphatidylcholine, and cholesterol) found in healthy animal tissues and other organisms in addition to *T. pallidum* and used in VDRL and RPR tests. The lipid composition of *T. pallidum* was first described in 1979 when it was reported that the organism contained all the phospholipids used in nontreponemal tests (*48*). Genomic analysis of *T. pallidum* further revealed the lack of certain enzymes for biosynthetic pathways necessary for these cytoplasmic and outer membrane phospholipids, indicating an inherent requirement for phospholipids from the host (*52*).

The increase in antibodies to cardiolipin, phosphatidylcholine, and cholesterol during *T. pallidum* infection is likely the result of a combination of antigens from both the bacteria and the host, not just from host tissue damage. In a rabbit model, *T. pallidum* cardiolipin induced a high antibody titer during active infection (*49*). Inoculating rabbits with inactivated *T. pallidum* resulted in a lower anticardiolipin titer, suggesting the increased response observed during active infection was attributable to immune stimulation from a combination of cardiolipin released from *T. pallidum* and damaged host cells (*49*). Because the antigens used in nontreponemal tests are found in *T. pallidum* membranes and host membranes, referring to these tests as nontreponemal is a misnomer. A 2019 study demonstrated that 11% of 526,540 reactive nontreponemal tests were not associated with syphilis, and in those cases, the tests were detecting antibodies to nontreponemal antigens generated by host tissue damage from other diseases (*53*). However, 89% of the reactive tests were associated with syphilis, implying that most nontreponemal tests detect antibodies triggered by *T. pallidum* phospholipid antigens during infection. Purported nontreponemal tests could more accurately be called lipoidal antigen tests. Hereafter in this report, these tests will be referred to as nontreponemal (lipoidal antigen) tests.

#### Treponemal Test

The term treponemal test was introduced in 1960 along with nontreponemal tests (*54*). Treponemal test remains an accurate description of a test that detects an antibody response to antigens specific to *T. pallidum*.

#### Nonspecific Antibodies

The term nonspecific antibodies has been used in the syphilis literature to characterize antibodies that are not specific to *T. pallidum* but are detected in nontreponemal tests. All antibodies bind to specific epitopes on an antigen and are specific to that antigen. However, the antibodies might not be specific for the detection of the disease or condition for which the test is ordered; thus, their presence affects the test specificity. Reporting antibody specificity and the effect on test specificity rather than using the blanket term nonspecific antibodies would be more accurate.

## Principles for Syphilis Diagnosis

Indications for syphilis testing include identification of individual, population, or community risk factors for exposure to *T. pallidum;* signs and symptoms suggestive of syphilis; or a known sexual contact of someone who has syphilis. The selection of laboratory tests and interpretation of results vary by stage of syphilis and previous treatment history. After diagnosis and staging has occurred, benzathine penicillin G is the recommended therapy for clinical resolution of infection and avoidance of sequelae (*55*). Patients with a history of penicillin allergy should be managed according to CDC’s *Sexually Transmitted Infections Treatment Guidelines, 2021* (*55*).

Testing for syphilis is based on the detection of reactive antibodies (typically in serum or cerebrospinal fluid [CSF]), suggestive of exposure to *T. pallidum;* direct observation of the organism by darkfield or fluorescent microscopy of lesion fluids or exudate; or histologic assessment of infected tissues or amplification of *T. pallidum-*specific nucleic acid sequences in fluids, exudate, or tissue biopsy material. Conventional microscopy used to examine Gram-stained smears is insufficient to visualize *T. pallidum* because of the bacterium’s slender morphology and poor uptake of aniline dyes (*51*). No available nucleic acid amplification tests (NAATs) are cleared by FDA for marketing in the United States, and culture for *T. pallidum* is cumbersome and is available only in selected research laboratories. Nontreponemal (lipoidal antigen) tests are most suitable for screening or diagnosis in conjunction with a medical history and physical examination when antibody titers are important to determine recent exposure to infection, a presumptive diagnosis in persons with signs or symptoms suggestive of syphilis, or to determine response to treatment.

Treponemal tests target specific *T. pallidum* antigens, either intact or sonicated *T. pallidum* or defined recombinant proteins; these tests were traditionally used to confirm that a reactive nontreponemal (lipoidal antigen) test is the result of *T. pallidum* infection (*51*). Treponemal antibodies generally persist after treatment and cannot be used to distinguish between a current infection or a previously treated infection. None of the nontreponemal (lipoidal antigen) or treponemal tests can distinguish infections caused by other *T. pallidum* subspecies. Multiple capillary whole blood immunoassays for which the specimen is collected by skin puncture have been developed as rapid tests and might offer diagnostic utility in clinical, public health, or nonclinical settings. Direct detection tests of *T. pallidum* are limited to darkfield microscopic examination of lesion fluids, staining of lesion fluid or exudate smears or tissue sections obtained by biopsy for treponemal spirochetes, or amplification of specific nucleic acid sequences by validated laboratory-developed tests.

## Recommendations for Syphilis Testing in the United States

### Nontreponemal (Lipoidal Antigen) Tests

**FIGURE 1:**
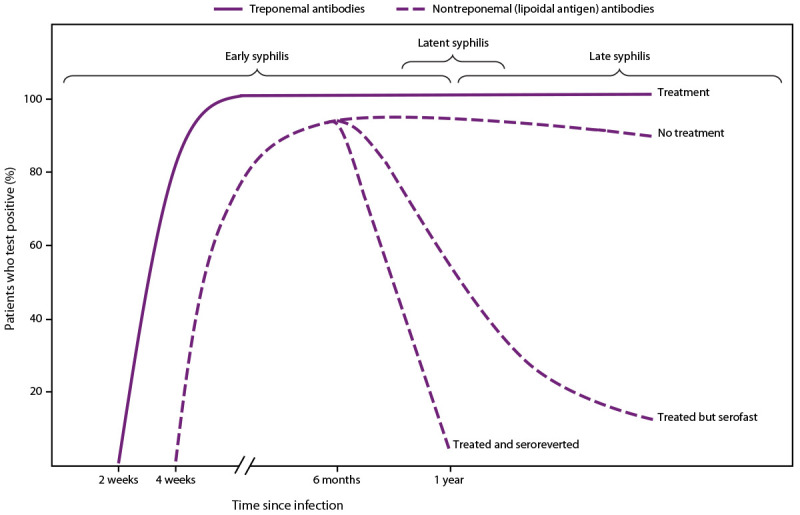
Serologic response to infection with *Treponema pallidum*, the causative agent of syphilis **Source:** Adapted from Peeling RW, Mabey D, Kamb ML, Chen X-S, Radolf JD, Benzaken AS. Syphilis. Nat Rev Dis Primers 2017;3:17073. Used with permission.

 Nontreponemal (lipoidal antigen) tests typically have been used as a screening test for syphilis, as a diagnostic test when patients have signs or symptoms suggestive of syphilis or have a known sexual contact, when assessing possible reinfections, and when monitoring treatment outcome (Figure 1). RPR and VDRL tests are still the primary screening methods used in public health laboratories in the United States (*56*); other FDA-cleared nontreponemal (lipoidal antigen) tests (e.g., the toluidine red unheated serum test [TRUST] and unheated serum reagin test [USR]) are available but are less commonly used in the United States. Regardless of which test method is applied, serum antibody titers from RPR, VDRL, and other nontreponemal (lipoidal antigen) tests should not be used interchangeably to manage patients because they are different test methods and the subjective titer results can vary by laboratory. Therefore, patient specimens should be tested using the same nontreponemal (lipoidal antigen) test method and specimen type.

The manual nontreponemal (lipoidal antigen) tests are flocculation tests that detect antibody-antigen complexes that fall out of solution as a precipitate. Microscopic or macroscopic procedures have been developed to detect the precipitate that forms after specific binding of antibodies to a combination of cardiolipin, cholesterol, and phosphatidylcholine that are used as antigens in nontreponemal (lipoidal antigen) tests. VDRL tests are read microscopically at 100x magnification (*51*). The RPR test uses charcoal to aid in detection of the flocculant, and the results can be read macroscopically because the antigen-antibody lattice traps the charcoal particles. The TRUST test uses toluidine red dye in place of charcoal.

Nontreponemal (lipoidal antigen) tests are usually performed manually; however, certain RPR tests have been automated for higher throughput. The automated systems digitally analyze the density and size of antibody-antigen flocculation and store results for future retrieval (*57*–*59*). Results from any nontreponemal (lipoidal antigen) test should be reported as an endpoint titer, and not with greater or less than values, to allow for optimal clinical interpretation. Certain automated RPR tests have a constrained serum dilution range (e.g., 1:40–1:64) that might be incapable of generating an endpoint titer beyond this range. In these situations, the titer range of the automated test must be considered, and specimens should require reflex testing using a manual RPR procedure to establish an endpoint titer at either the lower or upper bounds before reporting.

Whether automated or manual, performance depends on multiple factors, including specimen type and quality, stage of syphilis, presence of autoimmune or other diseases, and presence of infections or coinfections with organisms other than *T. pallidum*. Nontreponemal (lipoidal antigen) tests might be less sensitive than treponemal tests in early primary syphilis and tend to wane with time regardless of treatment. Before testing, test and specimen type should be carefully considered because serum and plasma cannot always be used interchangeably, and certain nontreponemal (lipoidal antigen) tests require heat treatment of specimens.

The subjective nature of results interpretation for manual tests as well as variability among laboratories and technicians pose challenges for clinicians who compare titers with stage of syphilis for treatment purposes, especially when assessing possible reinfection or monitoring treatment outcomes. One caveat of nontreponemal (lipoidal antigen) tests is that a reactive result could be a false positive because of recent conditions (e.g., infections, vaccinations or injection drug use, or underlying autoimmune or other chronic conditions). Nonetheless, when performed by an experienced laboratory technician and used in conjunction with treponemal tests, clinical history, physical examination, and contact history, the nontreponemal (lipoidal antigen) tests are a highly reliable testing method for screening and determining the endpoint titer for subsequent serologic monitoring posttreatment.

#### Serologic Response After Treatment

Nontreponemal antibody titers usually decrease at least fourfold during the 12 months after syphilis treatment (Figure 1), particularly among persons treated during the early stages of infection, and might become nonreactive over time, especially among patients treated before the secondary stage of syphilis (*60*–*62*). However, in certain persons, the decrease in nontreponemal antibody titers is less than fourfold despite recommended treatment. A prospective randomized, double-blind, multisite study of therapy for early syphilis (n = 541) found that 14% of patients had a less than fourfold serologic titer decline 12 months posttreatment; patients living with HIV infection who had primary or secondary syphilis were more likely to have an inadequate response than those without HIV infection (*60*). In addition, titers might not serorevert to a nonreactive result after treatment and remain persistently reactive, often referred to as the serofast state. This state is most common in persons treated ≥1 year after acquiring syphilis or in persons with multiple episodes of syphilis. Titers are typically ≤1:8, but higher titers also have been observed (*63*,*64*). Additional recommendations regarding clinical interpretation of nontreponemal titers are available in CDC’s *Sexually Transmitted Infections Treatment Guidelines, 2021* (*55*). Clinicians can consult with the STD Clinical Consultation Network for assistance with complex cases of titer interpretation (https://stdccn.org/render/Public).

**Recommendation for endpoint titers.** Endpoint titers (the highest dilution yielding a reactive result) should be determined and clearly reported when testing serum with nontreponemal (lipoidal antigen) assays that detect antibodies to lipoidal antigens (i.e., RPR and VDRL). Reports should not contain mathematical symbols such as > or < signs (Box).

BOX **CDC laboratory recommendations for syphilis testing, United States, 2024**

**Recommendation for endpoint titers.** Endpoint titers (the highest dilution yielding a reactive result) should be determined and clearly reported when testing serum with nontreponemal (lipoidal antigen) assays that detect antibodies to lipoidal antigens (i.e., rapid plasma reagin and Venereal Disease Research Laboratory). Reports should not contain mathematical symbols such as > or < signs.
**Recommendation for syphilis serologic testing algorithm.** Serologic tests that measure antibodies to both nontreponemal (lipoidal) and treponemal antigens related to syphilitic infections should be used in combination, when the primary test is reactive, to aid in the diagnosis of syphilis. Sole reliance on one reactive serologic test result can misclassify a patient’s syphilis status. Both the traditional syphilis screening algorithm (initial screening with nontreponemal [lipoidal antigen] assays) and the reverse syphilis screening algorithm (initial screening with treponemal immunoassays) are acceptable. The preferred algorithm should be based on laboratory resources, including staff, space and costs, test volume, and patient populations served.
**Recommendation for serologic syphilis testing.** Nontreponemal (lipoidal antigen) tests (e.g., rapid plasma reagin or Venereal Disease Research Laboratory) are not interchangeable when used to determine antibody titers; testing on follow-up samples must be performed with the same type of test. The *Treponema pallidum* particle agglutination test is the preferred manual treponemal test.
**Recommendation for syphilis serologic testing in pregnant persons.** Nontreponemal (lipoidal antigen) and treponemal tests should be interpreted in the same manner regardless of pregnancy status.
**Recommendation for syphilis serologic testing in persons living with HIV/AIDS.** Nontreponemal (lipoidal antigen) and treponemal tests should be interpreted in the same manner regardless of HIV status.
**Recommendation for the direct detection of *Treponema pallidum* by darkfield microscopy.** Darkfield microscopy should be maintained if already in use or established in sexually transmitted diseases clinics where a point-of-care test for primary or secondary syphilis diagnosis would be beneficial for timely patient treatment.
**Recommendation for direct detection of *Treponema pallidum* by immunohistochemistry and silver staining.** Immunohistochemistry is preferred over silver staining for formalin-fixed, paraffin-embedded tissue sections regardless of anatomic site.

**Comment and evidence summary.** Antibody titers measured by nontreponemal (lipoidal antigen) tests can correlate with infection status and are the only tests available to monitor treatment outcome (*60**,**62*). A fourfold change in titer between two results with the same nontreponemal (lipoidal antigen) tests is considered clinically significant (*55*). Titers need to be reported for appropriate clinical management. Serum samples tested with certain automated RPR tests that are outside the dilution range of the test should be reflex tested using a manual RPR.

#### Prozone

**FIGURE 2:**
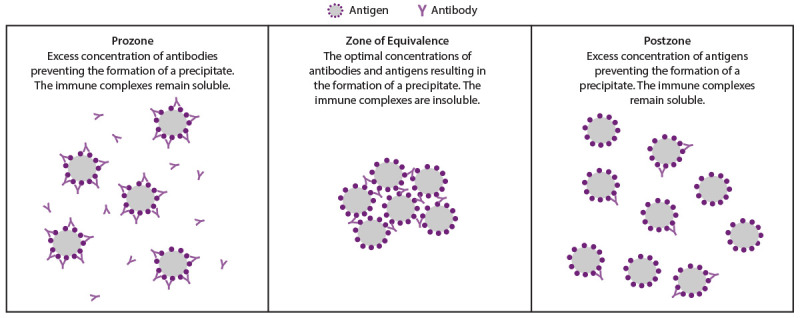
Effect of antibody and antigen concentration on agglutination

The detection of antigen-antibody interactions in agglutination or flocculation assays is dependent on the formation of antigen-antibody complexes that clump cells in agglutination tests or aggregates of small particles known as floccules. Many epitopes on an antigen can be bound by an antibody specific to the antigen. Immunoglobulin G (IgG) antibodies have two binding sites and immunogloubulin M (IgM) antibodies have 10 binding sites that can bind up to 10 identical antigens, respectively. As these interactions continue, a lattice structure can develop and become sufficiently large to cause agglutination or flocculation. The level of agglutination or flocculation varies depending on the relative concentrations of antigen and specific antibodies. Agglutination and flocculation assays standardize the antigen concentrations to maximize the formation of a lattice in a reactive test. Excess antibodies in serum or antigens in the assay can interfere with the development of a lattice if each antibody molecule binds to a single (instead of two) antigen epitope (Figure 2). In this case, cross-linking fails to occur and a lattice will not form, which can occur especially in an undiluted serum specimen. This false-negative phenomenon is referred to as a prozone or hook effect because it occurs before the zone of equivalence where the concentration of antibodies and antigens are sufficient for agglutination or flocculation. A prozone can be avoided if the serum sample is diluted before testing. False-negative results attributable to a prozone have been reported for nontreponemal (lipoidal antigen) but not for agglutination-based treponemal tests (*51*,*65*).

In two studies of 4,328 and 46,856 patients who had specimens referred for syphilis testing, false-negative RPR tests caused by a prozone were rare (<0.85%) (*65*,*66*). In one study, prozone in an RPR test occurred at all stages of syphilis but was more common during primary and secondary syphilis (4.7% and 1.8%, respectively) (*65*). Diluting serum can remove the prozone; however, no specific dilution values can ensure all effects of a prozone are removed. In the same study, among 36 serum samples with a prozone, 11 required serial dilutions from 1:8 to 1:16 to remove the prozone; 22 of these 36 samples required dilutions ranging from 1:32 to 1:128 for the optimal concentration of antibodies and antigens for agglutination (*65*). Two samples continued to have a prozone until they were diluted to 1:256 and one to 1:512. Because the prozone phenomenon is considered rare in a general population screened for syphilis, routinely diluting all nonreactive, undiluted nontreponemal (lipoidal antigen) tests is not recommended. However, laboratories should rule out a prozone using a dilution series for a nontreponemal (lipoidal antigen) test when requested by a clinician. A clinician should request a prozone rule out if a patient with signs or symptoms suggestive of syphilis has a nonreactive, undiluted nontreponemal (lipoidal antigen) test result or when unusual graininess is observed in the test of undiluted serum.

#### Biologic False Positive

A nontreponemal (lipoidal antigen) test that is reactive for conditions other than syphilis is referred to as a biologic false positive (BFP). Persons with antibodies that are reactive in the nontreponemal (lipoidal antigen) tests, but are nonreactive in a confirmatory treponemal test, are defined as BFP reactors. Health departments frequently retain records of persons with known BFP reactions; these data can assist clinicians in a future evaluation of possible syphilis infection in such persons. Reactive nontreponemal (lipoidal antigen) tests attributable to BFP have been estimated to occur in 0.2%–0.8% of the population and are associated with medical conditions other than syphilis (*67*–*71*). BFP reactions are attributable to other infections including malaria, leprosy, and HIV; recent vaccinations; autoimmune disorders; and injection drug use (*51*).

### Treponemal Tests

Treponemal tests are clinically used to confirm results of reactive nontreponemal (lipoidal antigen) tests and evaluate patients with signs suggestive of syphilis in early primary infection when nontreponemal (lipoidal antigen) tests might not yet be reactive. Treponemal tests can also be automated for high throughput screening in blood banks and in large laboratories for routine screening using the reverse sequence algorithm. Antibodies detected in treponemal tests typically persist for life despite treatment unless treatment occurs early in the course of infection; approximately 15%–25% of patients treated for primary syphilis can revert to a nonreactive treponemal test (FTA-ABS and MHA-TP) result within 2–3 years after treatment (*61*,*62*). In these two studies, no patients treated for secondary syphilis or stages of longer duration of infection seroreverted the reactive treponemal test. Seroreversion of treponemal tests can also occur in patients with advanced HIV disease and AIDS (*72*,*73*).

No published data are available that examined whether reversion to a nonreactive treponemal test occurs with an enzyme immunoassay (EIA) or a chemiluminescence immunoassays (CIA) after treatment for syphilis. Treponemal tests, unlike nontreponemal (lipoidal antigen) tests, cannot be used to monitor response to therapy because they remain reactive indefinitely. In patients with a history of treated syphilis and reactive treponemal test results, additional treponemal testing is not helpful for detecting reinfection and is not recommended. In this case, nontreponemal (lipoidal antigen) testing titers along with clinical history of syphilis, physical examination, and sexual risk assessment, including contact history, must be used to determine infection status.

Manual treponemal tests include FTA-ABS, TPPA, Captia Syphilis IgG EIA, Trep-Sure EIA, and Zeus Scientific EIA. Manual assays are typically used as reflex tests to confirm reactive nontreponemal (lipoidal antigen) specimens in the traditional testing algorithm. The FTA-ABS test is based on florescence microscopy and uses a fluorescein isothiocyanate-labeled antihuman immunoglobulin to detect antibody binding to whole *T. pallidum* that has been fixed on a glass slide. TPPA is an indirect agglutination assay with *T. pallidum* antigens bound to gelatin particles.

The manual TPHA and MHA-TP tests are no longer available for in vitro diagnostics in the United States but are still used in certain international settings. TPHA and MHA-TP are indirect agglutination with *T. pallidum* antigens bound to avian or ovine erythrocytes. MHA-TP is a microplate version of TPHA.

As of December 31, 2021, a total of 12 FDA-cleared automated treponemal immunoassays were available for clinical use, including EIA, CIA, and multiplex flow (microbead) immunoassays (MFIA). In contrast to the manual assays, the treponemal immunoassays are often run as the initial test in a reverse sequence screening algorithm. All FDA-cleared treponemal tests can be performed on serum; certain tests also can be performed on plasma, including heparin, EDTA, and citrate plasma. Certain laboratories also have also validated use of treponemal tests with dried blood spots (DBS); however, no available tests have been cleared by FDA for this specimen type, nor have data been published on DBS specimens collected in the United States to aid in the diagnosis of syphilis.

The reading output is typically an index value calculated as a signal to cutoff ratio (S/CO) or fluorescence ratio using values between the specimen and calibrator controls. Equivocal results should be retested according to algorithms in the package insert. The raw reading outputs and index values can be stored for future retrieval. The strength of the S/CO from immunoassays is an estimate of relative binding between molecules in the assay and has been researched as a predictor for positivity in hepatitis C and HIV confirmatory tests (*74*–*78*). When applied to treponemal immunoassays, multiple studies reported strong correlation between increasing index value strength and reactive results from an independent treponemal test or a combination of nontreponemal (lipoidal antigen) and treponemal tests, with most studies demonstrating 91%–100% correlation between S/CO cutoffs and TPPA positivity (*79*–*84*). Additional research is needed to establish test-specific cutoff values that are likely to be true positives for each of the FDA-cleared immunoassays. S/CO cutoff values could eliminate the need to adjudicate discrepant results between treponemal immunoassays and nontreponemal (lipoidal antigen) tests with a second TPPA.

For discordant nontreponemal (lipoidal antigen) and treponemal test results, an additional treponemal test is recommended using a different type of treponemal test assay and target (e.g., TPPA). Until further data are available regarding the role of S/CO cutoffs in a screening algorithm, the cutoff value could be an additional data point to assess likelihood of infection in complex situations (e.g., among pregnant persons with low risk for syphilis). Clinicians with these types of cases should contact the STD Clinical Consultation Network for assistance (https://stdccn.org/render/Public).

#### Blood Bank Screening

Blood donations are required to be tested for antibodies to *T. pallidum* as outlined in 21 CFR 610.40(a)(2). Persons that donate blood found to be serologically reactive are deferred (21 CFR 610.41[a]) and notified (21 CFR 630.40). Updated FDA recommendations for screening blood donors for syphilis are available at https://www.fda.gov/media/85283/download. The list of tests to screen blood donations for infectious agents is available at https://www.fda.gov/vaccines-blood-biologics/complete-list-donor-screening-assays-infectious-agents-and-hiv-diagnostic-assays.

### Traditional and Reverse Algorithms for Syphilis Screening

**FIGURE 3:**
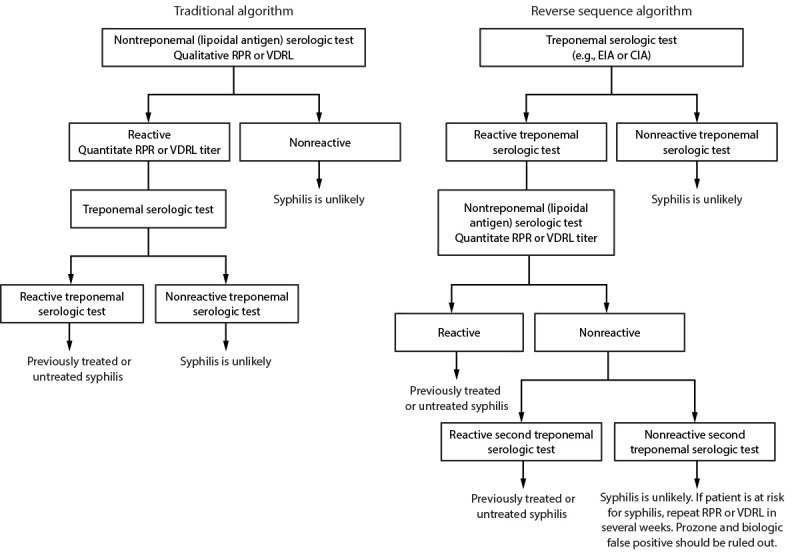
Algorithms that can be applied to screening for syphilis with serologic tests — CDC laboratory recommendations for syphilis testing in the United States, 2024 **Abbreviations:** CIA = chemiluminescence immunoassay; EIA = enzyme immunoassay; RPR = rapid plasma regain; TPPA = *Treponoma pallidum* particle agglutination; VDRL = Venereal Disease Research Laboratory.

The traditional algorithm for syphilis serologic screening begins with a nontreponemal (lipoidal antigen) test, and any reactive specimens are tested for confirmation by a treponemal test (Figure 3). This sequence has been widely used for decades because nontreponemal (lipoidal antigen) tests were relatively inexpensive and treponemal tests were manual, labor intensive, more costly, and limited in number. However, automated treponemal immunoassays, which were originally cleared by FDA for blood bank screening, are now cleared by FDA for clinical screening, leading to the reverse sequence algorithm. Initial screening with an automated treponemal test of a sample with a positive result must be followed by a quantitative nontreponemal (lipoidal antigen) test. When the reverse sequence algorithm is used, any discordant results should be adjudicated by a second treponemal assay (e.g., TPPA) that has a different format and includes different antigens (*85*).

The number of clinical laboratories performing traditional, reverse, or both algorithms was assessed among 2,360 laboratories participating in the 2015 College of American Pathologists (CAP) syphilis serology proficiency testing program in the United States (*86*). Of the 1,911 laboratories that responded, 81.1% (n = 1,550) offered only one algorithm, 9.5% (n = 181) offered different algorithms depending on patient demographics or clinician preference, and 9.4% (n = 180) reported being uncertain whether a single algorithm was offered. Approximately two thirds of laboratories (63.1%; n = 1,205) reported using the traditional algorithm, 15.9% (n = 304) reported using the reverse sequence algorithm, 2.5% (n = 47) reported using both algorithms, 5.9% reported that they did not know, and 3.9% reported “other.” Of responding laboratories, 8.8% (n = 169) stated that they did not reflexively perform a confirmation test. A 2017 survey by APHL reported that 58 of 73 (79.5%) public health laboratories used the traditional algorithm, and 20.5% used the reverse algorithm (https://www.aphl.org/aboutAPHL/publications/Documents/ID-2020Jan-2017-STD-Testing-Survey-Report.pdf). The CAP and APHL surveys should be updated to track changes in clinical laboratory practices over time.

A prospective comparison of 1,000 patient samples from a population with a low prevalence of syphilis tested with both algorithms found 15 (1.5%) that were reactive by the reverse sequence algorithm starting with the BioPlex IgG and four (0.4%) that were reactive by the traditional algorithm with RPR as the first test (*87*). The four samples that were reactive by RPR were confirmed to be positive by TPPA. The false-positive EIA rate (e.g., EIA reactive, RPR nonreactive, and TPPA nonreactive) was higher in the reverse sequence algorithm than the traditional algorithm (0.6% versus 0%). CDC reported a similar false-positive rate for treponemal immunoassay (0.6%; 866 of 140,176) when using the reverse sequence algorithm during 2006–2010 (*85*).

Data are conflicting regarding the cost-effectiveness of the traditional versus the reverse sequence algorithm. The traditional algorithm might be more cost-effective (lower cost per adverse event prevented) in settings with a low prevalence of syphilis (approximately 0.5%) and cost saving in higher-prevalence settings (approximately 10%) (*88*,*89*). These data are not consistent with a study that reported the reverse sequence algorithm as being cost-effective when applied to screening lower-prevalence prenatal and nonprenatal populations with a syphilis prevalence of 0.076% and 1.94%, respectively (*90*). In an economic impact model on a local sexually transmitted diseases (STD) program in Los Angeles County, California, the reverse algorithm was less expensive and identified more patients for treatment if the cost of the treponemal test was $1.67 less than the nontreponemal (lipoidal antigen) test cost of $5.80 (*91*). Testing, treatment, and follow-up costs were included in the analysis. Applying 2015 test costs from the 2015 CMS laboratory fee schedule in which treponemal tests costs were three times more costly than nontreponemal (lipoidal antigen) tests, the reverse sequence algorithm was more costly than the traditional algorithm. Each additional syphilis case detected would cost an estimated $1,242.17 when using reverse sequence algorithm with 2015 CMS test costs. These data highlight the need to consider local costs, including testing, treatment, and follow-up costs, when choosing the best algorithm for syphilis screening.

Each algorithm has advantages and disadvantages and both are acceptable (Table 1). The traditional algorithm might be less sensitive in detecting early or late latent syphilis, although an increase in false positives might occur when applying the reverse algorithm in low-prevalence populations (*22*). The development of antibodies that react with nontreponemal (lipoidal antigen) and treponemal tests might take up to 2 weeks after primary infection with *T. pallidum* (*92*,*93*) (Figure 1). The main advantage of automated treponemal immunoassays in high-volume laboratories is increased throughput and reduced labor costs. Considerations for test and algorithm selection include cost, labor, volume of specimen test requests, throughput, laboratory space, and turnaround time. In addition, clinicians and state and local public health STD programs need nontreponemal (lipoidal antigen) test results coupled with treponemal test results for timely clinical management and public health reporting. If one test result in the algorithm is delayed and needs to be coupled with the initial test by the clinician or the STD program, matching errors can occur, and clinical management and reporting can be delayed. The laboratory processing the initial screening test should ensure the second or third (if necessary) test results, especially if performed in a different laboratory, are linked with the screening test result when the report is sent to the ordering clinician and public health department.

**TABLE 1:** Comparison of traditional and reverse algorithms for syphilis screening by serology — CDC laboratory recommendations for syphilis testing, United States, 2024

Parameter	Traditional algorithm with a nontreponemal (lipoidal antigen) test as the initial test	Reverse algorithm with a treponemal test as the initial test
Reagent cost	Rapid and inexpensive reagents	Higher reagent cost per specimen Automated treponemal tests widely available with high throughput and lower human labor costs
Specimen throughput	Good for small-throughput laboratories Less suitable for high-throughput laboratories because of labor and resources needed and occupational hazard of pipetting of individual specimens	Possible batching of samples that could delay test result turnaround time
Performance characteristics of the first test in the algorithm	Results of nontreponemal (lipoidal antigen) tests can be subjective, and there is laboratory variability in titers Possible prozone reaction that might be falsely interpreted as negative unless the serum sample is diluted Biologic false-positive resulting from nonspecific reactivity resulting from conditions other than syphilis Might be less sensitive for detecting early and late/latent syphilis	Treponemal tests produce objective results No prozone reaction Detects antibodies against *Treponema pallidum* antigens Might have increased detection of patients with early syphilis
Screening applications	Good for populations with a high likelihood of previous syphilis	If algorithm is used in populations with a high likelihood of previous syphilis, an increased number of primary screening tests could be false positives*

* False positives are defined as being a reactive serum specimen during the initial treponemal serologic test that is nonreactive when reflex tested by a nontreponemal (lipoidal antigen) test and a second treponemal test.

**Recommendation for syphilis serologic testing algorithm.** Serologic tests that measure antibodies to both nontreponemal (lipoidal) and treponemal antigens related to syphilitic infections should be used in combination, when the primary test is reactive, to aid in the diagnosis of syphilis (Box) (Figure 3). Sole reliance on one reactive serologic test result can misclassify a patient’s syphilis status. Both the traditional syphilis screening algorithm (initial screening with nontreponemal [lipoidal antigen] assays) and the reverse syphilis screening algorithm (initial screening with treponemal immunoassays) are acceptable. The preferred algorithm should be based on laboratory resources, including staff, space and costs, test volume, and patient populations served.

**Comment and evidence summary.** Antibodies detected by nontreponemal (lipoidal) and treponemal antigen tests vary by the stage of syphilis, treatment status, and past infection that was treated (*92*). Results from both types of serologic tests are required to help diagnose the stage of syphilis. Both traditional and reverse syphilis testing algorithms are used in the United States (*86*) and have about 99% concurrence between the two approaches (*85**,**87*). The cost-effectiveness of the two algorithms might vary by laboratory setting (*88**–**91*) and need to be considered by individual laboratories.

### Serologic and CSF Antibody Specimen Collection and Storage

Serum, plasma, and CSF are specimen types that have been used in syphilis assays that detect antibodies against *T. pallidum*. This section provides general guidance because the information is summarized from various sources including product inserts and manuals on standard laboratory practices (*51*,*94*). Product inserts should be reviewed for optimal specimen type, transport, and storage because they vary by test. Health care providers should contact laboratories for additional information on sample volumes for collection if additional tests are to be performed.

#### Serum Collection Devices and Storage

Serum is the most common specimen used for syphilis serologic assays. Whole blood is collected by a trained phlebotomist using a vacutainer tube without an anticoagulant, coagulants, or a serum separator component. The use of vacuum tubes with serum separators or coagulants has not been widely evaluated with syphilis serology tests and should be avoided unless stated as an acceptable collection device in the test’s product insert. The volume of whole blood collected should be approximately 2.5 times the volume of serum required for the test. Approximately 1 mL of serum is enough to process both nontreponemal (lipoidal antigen) and treponemal syphilis serology tests, with extra reserved for repeat testing if needed. Collecting more serum should be considered if tests for conditions other than syphilis tests are requested. After collection of whole blood, the tube should be left undisturbed at room temperature for approximately 15–30 minutes to allow for clot formation. Vacutainer tube or other tubes containing whole blood should not be refrigerated because lower temperatures will increase clotting time. Serum can be aspirated if the clot has retracted or after centrifugation at 1,000–2,000 xg for 10 minutes. Serum should be transferred into a clean polypropylene tube for shipping or storage. Serum should be stored at 2°C–8°C (35.6°F–46.4°F) and tested within 5 days or frozen at ≤−20°C (−4°F) for longer storage. Serum should not be stored in frost-free freezers because the freeze-thaw cycles in these appliances are detrimental to the stability of frozen serum samples. However, recommended storage conditions vary among tests, and the product insert should be reviewed for up-to-date information. Samples should be free of hemolysis (https://www.cdc.gov/ncezid/dvbd/specimensub/hemolysis-palette.html), icterus, bacterial contamination, and lipemia. Serum should be aliquoted for storage to avoid repeated freeze-thaw cycles that could result in diminished antibody reactivity because of protein degradation and denaturation.

#### Plasma Collection Devices and Storage

Plasma is acceptable for certain qualitative and quantitative syphilis serologic assays. Whole blood is collected by a trained phlebotomist using a vacutainer tube with an anticoagulant, including EDTA-treated, citrate-treated, or heparinized tubes. The blood volume collected should be approximately 2.5 times the volume of plasma required. Approximately 1 mL is enough plasma to process both nontreponemal (lipoidal antigen) and treponemal syphilis tests, with extra reserved for repeat testing if needed. Cells are removed from plasma by centrifugation at 1,000–2,000 xg for 10 minutes. The supernatant plasma should be immediately transferred to a clean polypropylene tube and tested 1–5 days after collection, depending on the test. The time that plasma can be successfully stored is typically shorter than for serum, although storage conditions vary among tests and certain ones allow for longer-term storage of plasma if frozen. The product insert should be reviewed for up-to-date information. Samples should be free of hemolysis (https://www.cdc.gov/ncezid/dvbd/specimensub/hemolysis-palette.html), icterus, bacterial contamination, and lipemia. Plasma should be aliquoted for storage to avoid repeated freeze-thaw cycles that could result in diminished antibody reactivity by tests because of protein degradation and denaturation.

#### CSF Collection Devices and Storage

Only medical personnel qualified to perform lumbar puncture can collect CSF. Approximately 1 mL of CSF, placed into a clean polypropylene tube, is enough CSF for syphilis serologic testing, with extra remaining for repeat testing if needed. A larger volume of CSF might be required for additional tests (e.g., protein, cell count, Gram stain, or culture). If testing is delayed more than 4 hours, store the CSF sample at 2°C–8°C (35.6°F–46.4°F) for ≤5 days. After 5 days, CSF should be stored frozen at ≤−20°C (−4°F). Blood contamination, which could cause a false-positive result because of the presence of serum-derived antibodies rather than CSF-produced antibodies, should be avoided when collecting CSF specimens.

### Serologic and CSF Antibody Test Performance

#### Sensitivity of Serologic Tests for Primary Syphilis

Estimating the sensitivity of nontreponemal (lipoidal antigen) tests during primary syphilis is best assessed when direct detection of *T. pallidum* is used as the comparator test to ensure proper staging of syphilis for the analysis. The sensitivity of RPR when compared with darkfield microscopy of lesion exudate ranged from 48.7% to 76.1% (*95**–**101*); however, one study reported a sensitivity of 92.7% (n = 109 patients) (*102*) (Supplementary Table 1, https://stacks.cdc.gov/view/cdc/138288). VDRL had a similar sensitivity range (50.0%–78.4%) (*95**–**99**,**102**–**107*). One head-to-head comparison study of RPR and VDRL nontreponemal (lipoidal antigen) tests from 76 patients with primary syphilis confirmed by darkfield microscopy demonstrated a sensitivity of 48.7% and 50.0% for RPR and VDRL, respectively (*101*). Studies that used a NAAT to detect *T. pallidum* nucleic acid from a lesion swab and staged primary syphilis on the basis of clinical examination findings and a positive NAAT reported that nontreponemal (lipoidal antigen) test sensitivity ranged from 80% to 95% (*108*–*112*). Studies using NAAT as the reference standard rather than darkfield microscopy in lesions suggestive of primary syphilis suggest that nontreponemal (lipoidal antigen) tests might be more sensitive than previously thought.

The sensitivity of manual treponemal tests in primary syphilis has been estimated from studies that used reference standards such as darkfield microscopy (*95*,*102*,*113*–*115*), clinical findings (*116*–*118*), or stored serum collected from patients staged as having primary syphilis, although the criteria used to stage the disease were not fully described (*119*–*123*) (Supplementary Table 2, https://stacks.cdc.gov/view/cdc/138288). MHA-TP had a sensitivity of 53.0%, 72.5%, and 88.6% in studies that used darkfield microscopy as the reference standard (*102*,*113*,*118*). In studies that used stored sera collected from patients who were clinically classified as having primary syphilis, MHA-TP had a sensitivity of 45.9%, 64% and, 88.6% (*114*,*118*,*123*). A 2019 study involving 959 patients, 55 of whom were classified as having primary syphilis (on the basis of serology, physical findings, and positive or negative darkfield microscopy) reported a sensitivity of 78.2% (95% CI = 65.0%–88.2%) and 94.5% (95% CI = 84.9%–98.9%) for FTA-ABS and TPPA, respectively (*115*). Other studies with fewer patients, different reference standards, or both are more difficult to compare; sensitivities of FTA-ABS and TPPA have ranged from 88.4% to 100% and 86.2% to 100%, respectively, for primary syphilis (*102*,*113*,*114*,*117*,*118*,*122*–*127*).

Among the automated treponemal immunoassays, few published data are available on test performance stratified by stage. One study found similar sensitivity for the ADVIA Centaur, Bioplex 2200 Syphilis IgG, Diasorin Liaison, and Trep-Sure in primary syphilis compared with TPPA and FTA-ABS (*115*); however, another study of 52 patients found poorer sensitivity of Trep-Sure in primary syphilis (53.8%; 95% CI = 39.5%–67.8%) (*121*).

Nontreponemal (lipoidal antigen) and treponemal tests might not yet be reactive in certain persons with primary syphilis, particularly those with very recently appearing lesions. Using darkfield microscopy as the sole comparator will skew results toward lower sensitivities because persons with early lesions are more likely to have a positive test by darkfield microscopy and be seronegative. Lesions of longer duration might become negative by darkfield microscopy because of immune clearance, but these persons are more likely to be seropositive. NAATs might be positive in both early and older lesions because this test method is not dependent on visualization of motile organisms. Additional studies of genital, anal, and oral lesions using both darkfield microscopy and NAATs as the reference standard, including studies that assess age of lesions, are needed to better refine the sensitivity estimates of nontreponemal (lipoidal antigen) and treponemal tests for primary syphilis.

#### Sensitivity of Serologic Tests for Secondary Syphilis

In studies that classified secondary syphilis on the basis of clinical diagnosis that included rash, mucocutaneous lesions or patchy alopecia, mucous patches, or condylomata lata; clinical diagnosis with visualized spirochetes on darkfield microscopy; or clinical diagnosis with reactive nontreponemal (lipoidal antigen) and treponemal serology, the sensitivity of both RPR and VDRL was 100% (*96**–**99**,**101**,**103**,**105**,**128**–**131*) (Supplementary Table 1, https://stacks.cdc.gov/view/cdc/138288). Only two studies reported an RPR sensitivity of <100% (91% and 97.2%) (*99*,*101*).

The sensitivity of the treponemal assay, MHA-TP, for secondary syphilis ranged from 96% to 100%, except in one study that reported 90% sensitivity (*113*,*114*,*118*,*123*) (Supplementary Table 2, https://stacks.cdc.gov/view/cdc/138288). The estimated sensitivity of FTA-ABS was >92% with six of eight studies reporting 100% (*113*–*115*,*117*,*123*–*125*,*127*). Of the two studies that found sensitivity to be <100% (*115*,*124*), FTA-ABS sensitivity was reported to be 92.8% (95% CI = 85.7%–97.0%) and 95.0% (95% CI = 76.4%–99.1%). TPPA was 100% sensitive in five studies (*115*,*116*,*124*,*126*,*132*). Among the automated treponemal immunoassays, few published data are available on test performance stratified by stage; however, the sensitivity of five treponemal immunoassays (Liaison, TrepSure, Bioplex 2200, ADVIA Centaur, and INNO-LIA) was estimated at 100% for secondary syphilis in one study of 98 patients (*115*).

The sensitivity of both nontreponemal (lipoidal antigen) and treponemal tests approaches 100% because of higher antibody titers during the secondary stage of syphilis. A prozone might need to be ruled out in specimens from patients with suspected secondary syphilis that are nonreactive in nontreponemal (lipoidal antigen) tests. Because laboratorians typically do not know the patient’s stage of syphilis when the serologic specimen is submitted, clinicians should specifically request to assess for prozone when clinically indicated (e.g., in patients who have signs and symptoms of syphilis and nonreactive nontreponemal [lipoidal antigen] test results).

#### Sensitivity of Serologic Tests for Latent Syphilis

Data are limited on nontreponemal (lipoidal antigen) test performance in early latent and late latent stages of syphilis, with limited information regarding reference standards, previous treatment status, patient population risk for syphilis, and specific stage of latency (*128*–*131*,*133*–*135*). Furthermore, some international studies use different definitions of early and late syphilis than are used in the United States.

No studies involving RPR test performance for latent syphilis have been conducted in the United States. Two international studies conducted approximately 10 years ago and without stratification by duration of latency (i.e., early latent of <1 year versus late latent of >1 year) make estimates of sensitivities difficult (*128*,*134*). Three international studies on the performance of VDRL in cases of latent syphilis reported sensitivities that ranged from 82.1% to 100% for early latent syphilis of <1 year and from 63% to 66% for late latent syphilis of >1 year or of unknown duration; however, the studies were limited by small samples sizes (n≤72), making the results difficult to interpret (*129*,*131*,*133*) (Supplementary Table 1, https://stacks.cdc.gov/view/cdc/138288).

The sensitivity of the manual treponemal tests (FTA-ABS, TPPA, and MHA-TP) ranged from 94.4% to 100% for the diagnosis of early latent syphilis; a wider range for late latent syphilis than early latent syphilis (84.5%–100%) has been reported (*113*,*115*,*116*,*118*,*120*,*124*) (Supplementary Table 2, https://stacks.cdc.gov/view/cdc/138288). Among the treponemal immunoassays, sensitivity ranged from 95% to 100% for early latent syphilis and from 91.7% to 100% for late latent syphilis (*115*,*119*,*120*,*136*) (Supplementary Table 2, https://stacks.cdc.gov/view/cdc/138288). Although the sensitivity of treponemal tests is generally high for early latent and late latent syphilis, the range of sensitivities identified in these studies suggests that additional studies are needed in larger samples where the duration of infection is better characterized. The duration of latency is often difficult to pinpoint; certain patients staged as late latent could have unknown latency duration, whereas other patients classified as late latent could have recently acquired their syphilis infection. This misclassification of duration of infection could falsely elevate the syphilis test performance sensitivity in patients with late latent syphilis.

The sensitivity of nontreponemal (lipoidal antigen) tests decreases during latent syphilis of longer duration because the antibody detected by these test titers diminishes over time. Typically, treponemal tests remain reactive during latent syphilis.

#### Sensitivity of Serologic Tests for Tertiary Syphilis

Because tertiary syphilis is rare in the postantibiotic era, published data are very limited on the performance of serologic tests for diagnosis of tertiary syphilis (e.g., gummatous disease, late neurosyphilis, and cardiovascular syphilis); further studies are unlikely to be done. One study estimated the sensitivities of the FTA-ABS and VDRL at 70.6% and 47%, respectively, in 17 patients with tertiary syphilis (*133*), although the criteria for the stage of diagnosis were not stated. There were several studies that examined sensitivity of treponemal tests (Liaison CIA, Captia EIA, and FTA-ABS) for detection of cardiovascular syphilis. All studies estimated sensitivity to be 100%; however, sample sizes were extremely small (n = 1–21 cases) (*119*,*120*,*123*,*137*,*138*). The largest study of cardiovascular syphilis included 21 patients and found sensitivities of the MHA-TP and FTA-ABS were 89.5% and 100%, respectively (*114*). The sensitivity of nontreponemal (lipoidal antigen) tests varies from 47% to 64% during tertiary syphilis (*21*), whereas treponemal tests remain reactive.

#### Specificity of Serologic Tests

Reference standards for specificity analyses varied widely and included apparently healthy volunteers, antenatal patients, syphilis-negative blood donors who were not living with HIV infection, and patients clinically characterized as not having syphilis (from serum banks or on the basis of previous test results or chart review). Certain studies of treponemal test specificity also used results from a different treponemal test or a consensus of a panel of treponemal tests as the reference standard.

Few head-to-head studies compared the specificity of RPR with VDRL specificity on well-characterized specimens. A study of 500 antenatal serum samples found little difference in specificity between VDRL and RPR (two versus one false positive, respectively) (*139*). Another study among 200 blood donors found VDRL was slightly less specific than RPR (98.5%, with RPR as the gold standard) (*140*).

For manual treponemal tests, one study found the specificity of FTA-ABS to be 87% (n = 128 patients) (*141*), whereas the specificity ranges of FTA-ABS and TPPA (95%–100% and 94%–100%, respectively) were similar in older studies (*102*,*113*–*115*,*117*,*118*,*122*–*127*). The specificity of the FTA-ABS test can be limited by laboratory expertise and quality control measures. For these reasons and on the basis of the recent high-quality, head-to-head study demonstrating superior TPPA test performance characteristics, the manual serologic TPPA test is preferred over the serologic FTA-ABS test. However, the CSF FTA-ABS can still help in excluding a neurosyphilis diagnosis because of its negative predictive value when performed in a laboratory experienced in the off-label use of this test. The immunoassays demonstrated specificity ranging from 94.5% to 100% (*119*–*121*,*137*,*142*–*149*); however, Trep-Sure was 82.6% (95% CI = 78.4%–86.1%) specific, significantly lower than the other immunoassays evaluated in a single head-to-head study of 959 patients (*115*).

**Recommendation for serologic syphilis testing.** Nontreponemal (lipoidal antigen) tests (e.g., RPR or VDRL) are not interchangeable when used to determine antibody titers; testing on follow-up samples must be performed with the same type of test (Box). The TPPA test is the preferred manual treponemal test.

**Comment and evidence summary.** Sensitivity and specificity estimates of RPR and VDRL were similar but not exact in head-to-head studies and studies that used similar reference standards (*95**–**99**,**101**–**104**,**106**–**108**,**111**,**112**,**139*). When assessing changes in antibody titers using nontreponemal (lipoidal antigen) tests, it is critical that the same test be used because titers are used by clinicians to classify the infection status of a patient and follow treatment response (*55*). A recent study with 959 patients estimated the sensitivity of FTA-ABS and TPPA to be 78.2% and 94.5%, respectively, when testing specimens from patients with primary syphilis (*115*). Two studies that tested specimens from patients with secondary syphilis reported a sensitivity of 92.8%–95.0% compared with 100% for TPPA (*115*,*124*). Many automated treponemal immunoassays are similar in sensitivity, and certain ones are slightly less specific when compared with the manual TPPA, except for the Trep-Sure test which has inferior specificity. Among the other immunoassays, data are insufficient to recommend one assay based on test performance.

#### CSF Antibody Tests for Neurosyphilis

Challenges associated with the diagnosis of neurosyphilis include a lack of consensus on the clinical implications of abnormal CSF findings in patients with no neurologic symptoms or signs but with serologic evidence of syphilis and poor distinction between asymptomatic and symptomatic patients in studies evaluating laboratory tests to aid in the diagnosis of neurosyphilis. In addition, the wide variation in reference standards that included CSF VDRL, CSF protein elevation and pleocytosis, CSF NAAT, CSF FTA-ABS, or other CSF treponemal and nontreponemal (lipoidal antigen) tests, limited direct comparisons of CSF antibody test performance among neurosyphilis studies. Finally, the CSF VDRL is the only FDA-cleared test recommended to aid in the diagnosis of neurosyphilis. Although no treponemal test is FDA cleared to aid in the diagnosis of neurosyphilis, the CSF FTA-ABS has been used off-label for years in unique clinical circumstances for its negative predictive value (e.g., in patients with nonspecific neurologic signs or symptoms, reactive serologic tests, and a negative CSF VDRL, even if CSF lymphocytic pleocytosis and elevated CSF protein are present).

Because asymptomatic or symptomatic CNS invasion can occur in persons with primary, secondary, latent, or tertiary disease, serum examination can confirm the presence of syphilis but does not address CNS invasion or involvement. Examination of CSF is required to confirm CNS invasion but is only recommended in patients with reactive serologic tests and signs or symptoms suggestive of neurosyphilis; the clinical significance of CSF laboratory abnormalities in patients without any neurologic findings is unknown (*55*).

#### Nontreponemal (Lipoidal Antigen) Tests for Neurosyphilis

Manual nontreponemal (lipoidal antigen) tests have been used to test CSF as an adjunct in cases of neurosyphilis, but performance estimates can vary widely depending on the reference standard. In three studies with a reference standard of detection of *T. pallidum* nucleic acid by NAAT on CSF, hearing or vision loss or neurologic signs and symptoms suggestive of neurosyphilis with a reactive CSF TPPA, or presence of at least 10 white blood cells in CSF and a positive CSF TPPA, sensitivity and specificity of CSF VDRL ranged from 66.7% to 85.7% and 78.2% to 86.7%, respectively, in 149–154 patients with neurosyphilis symptoms (*150*,*151*) (Supplementary Table 4, https://stacks.cdc.gov/view/cdc/138288). In these studies, CSF RPR sensitivity and specificity was 51.5%–81.8% and 89.7%–90.2%, respectively (*150*,*151*). CSF VDRL is the only FDA-cleared test to aid in the diagnosis of neurosyphilis.

Another study using a reference standard of reactive CSF FTS-ABS, increased CSF protein of >45 mg/dL, and CSF pleocytosis of ≥10 cells/mm^3^ estimated the CSF VDRL sensitivity in eight patients with symptomatic neurosyphilis to be 87.5% (*152*). The study did not report CSF VDRL specificity stratified by asymptomatic and symptomatic neurosyphilis; however, the combined specificity was 99%. The sensitivity of CSF RPR in this study was estimated to be 100% in symptomatic patients. The combined specificity estimate for CSF RPR was 99.3%. No data are available for the performance of automated nontreponemal (lipoidal antigen) RPR tests on CSF samples. Additional head-to-head studies with comparable high-quality, agreed-upon reference standards and well-characterized patient symptom status are needed to better understand CSF nontreponemal (lipoidal antigen) test performance.

#### Treponemal Tests for Neurosyphilis

The lack of a definitive diagnosis standard makes it difficult to interpret studies of the use of treponemal tests to support neurosyphilis diagnosis. Studies of treponemal test sensitivity in CSF included patients with symptomatic and asymptomatic neurosyphilis; various laboratory tests were used for the reference standard, including CSF white blood cell count, protein, and CSF-VDRL (*153*). Studies of test specificity included patients without syphilis as well as patients with syphilis but no symptoms suggestive of neurosyphilis. The variation in reference standards limits the ability to compare sensitivity and specificity estimates among studies. No CSF treponemal antibody tests are cleared by FDA to aid in the diagnosis of neurosyphilis.

Thirteen studies describing CSF FTA-ABS test performance were summarized in a previous systematic review (*154*). Sensitivity varied depending on whether the reference standard required reactive CSF-VDRL to meet the case definition (definitive neurosyphilis) or a combination of other criteria (presumptive neurosyphilis), including reactive nontreponemal (lipoidal antigen) or treponemal CSF, other CSF indices (pleocytosis or elevated protein), rabbit inoculation, or clinical signs and symptoms.

In studies of definitive neurosyphilis, sensitivity of CSF FTA-ABS was 90.9%–100% (*155*–*157*). In the two largest studies of presumptive neurosyphilis (n = 60 and n = 156), CSF FTA-ABS demonstrated 100% sensitivity (*158*,*159*).

CSF FTA-ABS specificity varied greatly depending on whether true negatives were patients without syphilis or patients with syphilis but not symptomatic neurosyphilis. Six studies included patients without syphilis as true negatives, and CSF FTA-ABS specificity was 100%. In 11 studies that included patients with syphilis but not symptomatic neurosyphilis, the specificity ranged from 55% to 100% (*154*), likely because of passive diffusion of serum antibodies across an inflamed blood-brain barrier. This wide range of specificity in patients with syphilis but without neurologic symptoms could lead to false-positive results and overtreatment in these patients and in patients with nonspecific neurologic symptoms where the diagnosis of neurosyphilis is unlikely. A negative CSF FTA-ABS result can be clinically helpful to exclude neurosyphilis in complex cases where the cause of nonspecific neurologic signs or symptoms is most likely from other conditions.

Data are limited on the use of CSF TPPA in public health and commercial laboratories, and no studies have been published on the performance of automated treponemal immunoassays in CSF. For CSF TPPA, three studies reported sensitivities of 75.6%–95.0%; the highest sensitivities ranged from 83.3% to 95.0% when a reactive CSF-VDRL was the reference standard for neurosyphilis (*160*–*162*). CSF TPPA specificity increased from 75.6% to 93.9% with increasing CSF TPPA titers from ≥1:160 to ≥1:640, respectively, when neurosyphilis was defined as a reactive CSF-VDRL or as new vision or hearing loss (*162*) (Supplementary Table 5, https://stacks.cdc.gov/view/cdc/138288). On the basis of these limited data, CSF TPPA might have similar sensitivity performance to CSF FTA-ABS in studies of patients with definitive or presumptive symptomatic neurosyphilis (*55*). However, further studies on CSF TPPA test performance and titers are needed before this treponemal test can be recommended for off-label use in unique clinical situations to aid in the diagnosis of neurosyphilis.

#### CSF Antibody Tests for Ocular Syphilis and Otosyphilis

Ocular syphilis and otosyphilis diagnoses are difficult, and data are limited on CSF nontreponemal (lipoidal antigen) and treponemal test performance in these clinical scenarios. Existing studies are largely retrospective with small sample sizes (<50) and use of CSF VDRL testing, with low sensitivity for both ocular syphilis (<50%) and otosyphilis (<10%) when compared with clinical manifestations and serological evidence of syphilis as reference standards (*163*–*173*). CDC’s *Sexually Transmitted Infections Treatment Guidelines, 2021* state that CSF analysis, including a cell count, protein determination, and CSF-VDRL, might be helpful in diagnosis of suspected ocular syphilis for patients without neurologic symptoms and no evidence of ocular infection on examination; however, it is not recommended in suspected otosyphilis among persons with isolated auditory symptoms and a normal neurologic examination (*55*).

No published data are available on CSF treponemal test performance in ocular syphilis, and limited studies of CSF treponemal tests in patients with otosyphilis include insufficient sample sizes and unsuitable reference standards. No CSF treponemal tests are recommended for off-label use in patients with suspected ocular syphilis or otosyphilis and no symptoms or signs suggestive of neurosyphilis.

#### Serologic Tests for Congenital Syphilis

Passive transfer of maternal antibody can cause positive treponemal test results in neonates and infants for >1 year (*174*). Performing a treponemal test (i.e., TPPA, FTA-ABS, or immunoassay) on neonatal serum is not currently recommended because interpreting these results is difficult (*55*). Although studies have found good correlation between IgM FTA-ABS or ELISA and clinical congenital syphilis findings or other reactive serology in neonates (*175*,*176*), these studies were not performed with commercially available IgM tests. No IgM test is recommended to aid in the diagnosis of congenital syphilis. Quantitative nontreponemal (lipoidal antigen) tests (e.g., RPR or VDRL) are recommended for use in newborns born to mothers with positive syphilis serologies during pregnancy (*55*). Nontreponemal (lipoidal antigen) tests should be performed on serum and not umbilical cord blood because umbilical cord blood can become contaminated with maternal blood and yield a false-positive result, and Wharton’s jelly within the umbilical cord can yield a false-negative result (*55*). The same nontreponemal (lipoidal antigen) test should be used for the infant that was used for the mother at delivery so titer levels can be compared (*55*).

#### Serologic Test Performance in Pregnant Persons

A 1995 study evaluating RPR serologic testing of 265 specimens from obstetric patients immediately after delivery demonstrated a sensitivity and specificity of 100% and 97.6%, respectively, when using clinical diagnosis and FTA-ABS, Captia Syphilis G, or both as reference standards (*177*). Similar to the low incidence of biologic false positives in the general population (<0.85%) (*65*), false positives are low among pregnant persons (0.6%); all initial reactive nontreponemal (lipoidal antigen) tests should be reflexed to a confirmatory treponemal antibody test (*66*).

Treponemal test performance data during pregnancy are limited. In a single study that included 2,000 patients, manual treponemal test specificity using concordance among both tests as the reference standard (e.g., FTA-ABS or TPHA) was high for both tests (99.8% and 99.95%, respectively); however, for pregnant persons, this study did not have a control group (*178*). For manual treponemal immunoassays, one study of Captia EIA used TPPA as the reference standard and included 9,896 pregnant patients and 24,346 nonpregnant persons who were screened at an institution that screens high-prevalence populations, including persons living with HIV infection and men who have sex with men (MSM) (*179*). Discordant immunoassay results (e.g., EIA positive, RPR negative, and TPPA negative) were more common for pregnant than nonpregnant persons (71.4% versus 43.5%). This is likely related to the lower prevalence of syphilis among pregnant persons screened compared with nonpregnant persons at higher risk screened. A retrospective study of aapproximately 100,000 pregnant persons screened with an automated immunoassay found 194 women with discordant immunoassay results; 156 of these women had a reactive Liaison CIA result, nonreactive RPR, and nonreactive TPPA (isolated CIA reactive), and 38 women had a reactive Liaison CIA, nonreactive RPR, and reactive TPPA (*180*). Among 77 women with an isolated CIA-reactive result who were retested by their provider, 41 (53%) seroreverted to nonreactive within 12 months.

**Recommendation for syphilis serologic testing in pregnant persons.** Nontreponemal (lipoidal antigen) and treponemal tests should be interpreted in the same manner regardless of pregnancy status (Box).

**Comment and evidence summary.** On the basis of existing data, treponemal tests perform no differently in pregnant persons and should be interpreted in the same manner as for nonpregnant persons (*177*,*179*,*180*). However, because of the lower prevalence of syphilis in pregnant persons in many areas of the United States, discordant immunoassay results identified with the reverse sequence screening algorithm need to be adjudicated with a treponemal test such as the TPPA and managed according to CDC’s *Sexually Transmitted Infections Treatment Guidelines, 2021* (*55*). False-positive nontreponemal (lipoidal antigen) tests in pregnancy occur at a similar rate to the general population (*65*,*66*).

#### Serologic Test Performance in Persons Living with HIV/AIDS

Data are limited on nontreponemal (lipoidal antigen) test performance for persons with HIV infection as a distinct group; most studies report RPR and VDRL sensitivity in general populations that include HIV-positive persons or HIV infection in the context of neurosyphilis or syphilitic posterior uveitis. A 2007 cross-sectional study of 868 patients with genital ulcer disease indicated that RPR test sensitivity and specificity for patients with HIV infection was 81.8% and 90.6%, respectively, which was comparable to results observed for the cohort without HIV infection (*181*). In addition, a 2017 study found no statistically significant difference in sensitivity or specificity on the basis of HIV infection status when evaluating 571 specimens using CSF VDRL and CSF polymerase chain reaction (PCR) with clinical neurologic symptoms as reference standards (*162*); using laboratory and clinical diagnostic criteria, CSF-VDRL sensitivity ranged from 49% to 68% and specificity ranged from 90% to 91%. Other studies of populations with varying levels of HIV prevalence found overall sensitivities of 72.5%–85% for serum RPR, 68.8% for CSF RPR, 13.3%–62.5% for CSF VDRL, and 72.6%–91.2% for serum VDRL (*95*,*152*,*163*,*169*,*182*).

Although data suggest that nontreponemal (lipoidal antigen) test performance sensitivities do not significantly differ between persons living with and without HIV infection, studies have reported increased likelihood of BFP in HIV-positive persons. In studies with samples sizes that ranged from 789 to 300,000, serum testing by VDRL or RPR indicated that the rate of BFP results was 2.5–34.5 times higher among HIV-positive persons than HIV-negative persons (*67*–*69*,*183*,*184*). These studies were conducted in populations before antiretroviral therapy was widely available or in populations where viral load was not assessed. BFP rates in persons living with HIV infection who are virally suppressed have not been studied.

Treponemal test positivity generally persists after previously treated infection, unless the infection is treated before the secondary stage, as has been previously described in persons without HIV infection. Before modern antiretroviral therapy, seroreversion of either the MHA-TP or FTA-ABS test was found to vary by severity of HIV disease in two studies and was lower for asymptomatic HIV infection (five of 69 patients) than symptomatic HIV/AIDS (eight of 21 patients) in one study (*62*). In another study, seroreversion was identified in 14% of 29 patients with asymptomatic HIV infection and 41% of 29 patients with symptomatic HIV infection (*72*). However, two subsequent studies including 31 and 104 patients found no difference in seroreversion of treponemal tests by HIV status in patients previously treated for syphilis (*113*,*185*). In a more recent study of 294 patients with previous syphilis followed for ≥6 months after treatment and with no signs of syphilis during the follow-up interval, 87% were reactive for FTA-ABS, 92% for TPPA, and 96%–99% for one of four treponemal immunoassays (*115*). Treponemal immunoassays were statistically significantly more likely to remain reactive compared with FTA-ABS (*115*).

**Recommendation for syphilis serologic testing in persons living with HIV/AIDS.** Nontreponemal (lipoidal antigen) and treponemal tests should be interpreted in the same manner regardless of HIV status (Box).

**Comment and evidence summary.** On the basis of existing data, nontreponemal (lipoidal antigen) and treponemal tests should be interpreted the same for patients with and without HIV infection (*95*,*115*,*152*,*162*,*181*).

### Direct Detection Tests for *T. pallidum*

#### Darkfield Microscopy

Darkfield microscopy has been the most widely used direct detection method for *T. pallidum,* but over time, has become less widely available in the United States as the health care delivery system has evolved (*56*,*186*). Darkfield microscopy is a morphology- and motility-based test that relies on examining live treponemal spirochetes and must be performed within 20 minutes of specimen collection (*51*,*94*). The test is useful for moist lesions of suspected anogenital primary or suspected secondary syphilis where treponemal spirochetes can be readily found (e.g., ulcerative lesions and condylomata lata). Suspected lesions of the external and internal genitalia (including the cervix) and rectum can be examined if serous fluid is collected according to established procedures for darkfield microscopy specimen collection (*51*). Darkfield microscopy on oral lesions is difficult to interpret because of the presence of oral commensal treponemes, which are easily confused with *T. pallidum*; therefore, it is not recommended to use darkfield microscopy on oral lesions.

An optimal specimen for darkfield microscopy is serous fluid that is free of red blood cells and collected on a microscope slide by using a touch preparation or sterile bacteriological loop. The lesion should be gently cleaned and abraded with a sterile gauze pad or a swab dipped in saline. Serous fluid will appear when slight pressure is applied to the base of the ulcer. A microscope slide should be used to collect the exudate, and a coverslip should be applied in a manner that avoids trapping air bubbles. Alternatively, a sterile bacteriological loop can be used to transfer the exudate to a slide. For cervical, intravaginal, and rectal lesions, serous fluid specimens can be collected with a moist swab and transferred to a glass slide.

Darkfield microscopic capability should be maintained or established in clinics in areas with a high prevalence of syphilis; rapid onsite detection of primary syphilis results in timelier treatment that benefits both patient care and public health. A well-trained microscopist and a darkfield microscope are required onsite so the sample can be examined within 20 minutes of collection before motility is compromised. Proficiency testing of darkfield microscopy should be ongoing, and training is provided by the National Network of STD Clinical Prevention Training Centers (https://www.nnptc.org). The use of commensal *Treponema refringens* and *Treponema denticola* for darkfield microscopy training is not recommended because these spirochetes can easily be confused with *T. pallidum* (*51*). Proficiency with darkfield microscopy requires the ability to distinguish *T. pallidum* from other commensal spirochetes on the basis of motility and morphology.

The sensitivity and specificity of darkfield microscopy, defined by clinical presentation and laboratory findings (i.e., serology or PCR), ranges from 75% to 100% and 94% to 100% for primary lesions and 58% to 71% and 100% on secondary lesions, respectively (*141*,*187*–*191*). Because serologic tests can be negative in early infection, darkfield microscopic examination of anogenital lesions suspected of being primary syphilis can result in a definitive diagnosis (*186*). The variation in darkfield microscopy sensitivity for primary lesions might be related to the duration of the lesion because most studies do not assess the age of the lesion when conducting performance studies for primary syphilis. Darkfield microscopy can still be used as a POC test for definitive diagnosis in any patient with anogenital lesions suggestive of primary syphilis.

The sensitivity of serology at the secondary stage of syphilis in adults is superior to darkfield microscopy; therefore, darkfield microscopy is not routinely recommended in suspected secondary syphilis, except for condylomata lata when POC serology is not available or negative and a definitive diagnosis is warranted. If available, darkfield testing also might be useful for testing moist lesions of congenital syphilis (e.g., bullous rashes and snuffles). The sensitivity of darkfield microscopy compared with rabbit infectivity testing (previous gold standard) on amniotic fluid for congenital syphilis diagnosis varies from 42% to 86% with a specificity of 100% (*192*,*193*). Because data are limited, darkfield testing on amniotic fluid is generally not recommended.

Commensal treponemes found in the oral cavity might be misinterpreted as *T. pallidum* (*51*); therefore, darkfield microscopy is not recommended for oral lesions. Darkfield microscopy is not recommended for CSF, lymph node aspirate, and other body fluids because scientific evidence for use with these specimen types is lacking. A list of test performance, specimen types, storage, and transportation-related guidance for direct detection syphilis tests is provided (Table 2) (Supplementary Table 6, https://stacks.cdc.gov/view/cdc/138288).

**TABLE 2:** Specimen types, storage, and transport for direct detection tests for *Treponema pallidum* — CDC laboratory recommendations for syphilis testing, United States, 2024

Direct detection test	Specimen types	Specimen storage and transport
Darkfield microscopy	Serous exudate of moist lesions (except oral lesions) should be collected directly on a microscope slide or using a sterile bacteriological loop; avoid red blood cells	Fresh, room temperature (20°C to 26°C; 68°F to78.8°F)
Immunofluorescent antibody test staining	Smear from suspected lesion(s)	Fresh, room temperature (20°C to 26°C; 68°F to 78.8°F)
Immunohistochemistry staining	Formalin-fixed and paraffin-embedded tissue sections of brain, placenta, umbilical cord, or skin lesions from secondary or tertiary syphilis	Room temperature (20°C to 26°C; 68°F to 78.8°F)
Silver stain	Formalin fixed and paraffin embedded tissue sections of brain, placenta, umbilical cord, or skin lesions from secondary, tertiary, or congenital syphilis	Room temperature (20°C to 26°C; 68°F to 78.8°F)
Nucleic acid amplification test	Primary syphilis: Serous exudate of moist lesions should be collected with a sterile Dacron swab and placed in a commercial transport medium Secondary syphilis: Mucous patches and condyloma lata specimens should be collected with a sterile Dacron swab and placed in a commercial transport medium Fresh frozen tissue biopsy or formalin-fixed and paraffin-embedded tissue Neonatal whole blood or serum; whole blood should be collected in an EDTA (purple top) tube	Frozen (−20°C to −80°C; −4°F to −112°F), frozen ice packs or dry ice

**Recommendation for the direct detection of *T. pallidum* by darkfield microscopy.** Darkfield microscopy should be maintained if already in use or established in STD clinics where a POC test for primary or secondary syphilis diagnosis would be beneficial for timely patient treatment (Box).

**Comment and evidence summary.** The sensitivity of darkfield microscopy in detecting *T. pallidum* from primary lesions ranges from 94% to 100% and 81% to 100% from secondary lesions when compared with NAATs (*141*,*187*–*191*). Darkfield microscopy can be more sensitive than serologic tests at the primary stage and offers the advantage of timely detection and rapid treatment of primary syphilis (*186*). The procedure is classified as moderately complex by CLIA, and the settings implementing the darkfield microscopy will require CLIA certification for such a test.

#### Immunofluorescent Antibody Staining for *T. pallidum* Detection

The direct fluorescent antibody test for *T. pallidum* (DFA-TP) method uses fluorescence-tagged specific antibodies to visualize *T. pallidum* in specimens from primary and secondary syphilis lesions. This test specimen collection method is similar to darkfield microscopy, except that after being placed on the microscope slide, the specimen is fixed and sent to a laboratory for processing. Generally, the DFA-TP test is equivalent in sensitivity to darkfield microscopy (*188*,*190*); however, whereas darkfield test performance to assess motility might decline with time, DFA-TP might be more sensitive in older primary lesions. DFA-TP also has the advantage of not requiring motile organisms to detect *T. pallidum*, and the reading of the results is more objective. The main disadvantages are that results take 1–2 days because they must be processed in a laboratory, and the commercial, FDA-cleared DFA-TP test is no longer available in the United States (*194*). Fluorescence-tagged monoclonal or polyclonal antibodies are commercially available but are not FDA cleared. For use in diagnostics and standard clinical laboratory practice, these reagents would need to be validated for clinical diagnostic testing and routine quality control would need to be performed.

#### Immunohistochemistry and Silver Staining

Immunohistochemistry (IHC) and silver staining are direct detection methods that have been used to stain and examine formalin-fixed, paraffin-embedded (FFPE) tissue biopsies from the skin, brain, placenta, umbilical cord, or other tissues. Biopsies can help identify the cause of atypical ulcers or skin lesions or those that do not respond to initial therapy (*55*). Silver staining (e.g., Warthin-Starry and Steiner stains) is a morphology-based test, whereas IHC is both immunologically and morphology based.

For IHC, the peroxidase-conjugated avidin-biotin complex (ABC) technique has been the most frequently evaluated method for tissue sections. The method involves heat-induced epitope exposure and incubation with rabbit anti-*T. pallidum* immunoglobulin antibodies. Subsequently, biotinylated anti-rabbit immunoglobulin antibodies are added, followed by incubation with peroxidase-conjugated ABC and visualization of the stained treponemal spirochetes. The main difference between the indirect immunofluorescence (IIF) method and IHC ABC is that the secondary antibody is labeled with a fluorescent dye in IIF.

Compared with a clinical or serological diagnosis of secondary syphilis, the IHC ABC method demonstrated 100% specificity across four studies, with sensitivity ranging from 64% to 94% (*187*,*191*,*195*,*196*). In one of these studies, the sensitivity of IHC ABC was compared with IIF on 37 tissue samples; the sensitivity was 95% and 89%, respectively (*191*).

The sensitivity of silver staining of FFPE skin biopsies reported in four studies ranged from 0% to 41% compared with darkfield microscopy, clinical diagnosis and stage of syphilis, and serology (*195*–*198*). Although specificity was not addressed in these studies, others reported challenges with interpreting stained sections because background staining of artifacts and reticulum fibers in skin tissue made it difficult to visualize treponemal spirochetes (*196*,*199*). Another study evaluated silver staining and an IIF assay on FFPE tissue sections from 17 cases of fetal death attributable to congenital syphilis and found the test sensitivities were 41% (seven of 17) and 88% (15 of 17), respectively (*200*). Because of both low sensitivity and challenges with distinguishing spirochetes, use of silver staining for direct detection of *T. pallidum* is no longer recommended for any type of FFPE tissue specimens (*195*).

IHC ABC should be used for evaluating atypical lesions and tissue biopsies for suspected syphilis (primary, secondary, congenital, and gummatous) when the diagnosis remains uncertain. Polyclonal antibodies used with IHC ABC might cross-react with intestinal or other spirochetes (e.g., *Borrelia burgdorferi*) (*196*,*201*). Further studies comparing the test performance of IIF with IHC ABC are needed.

For congenital syphilis testing, placenta and umbilical cord samples should be tested with the IHC ABC technique or IIF but not with silver stain. Placenta tissue samples should be taken at the periphery and close to where the cord is attached. A cord sample approximately 3–4 cm long should be obtained from a section distal to the placenta soon after delivery; the tissue should not be cleaned with antimicrobial-containing solution before sample collection (*201*). Tissue samples should be fixed in 10% buffered formalin at room temperature immediately upon collection and sent to a pathology laboratory for paraffin embedding and sectioning.

**Recommendation for direct detection of *T. pallidum* by immunohistochemistry and silver staining.** IHC is preferred over silver staining for FFPE tissue sections regardless of anatomic site (Box).

**Comment and evidence summary.** The sensitivity of IHC ranged from 64% to 94% (*187*,*191*,*195*,*196*), whereas silver stain had a sensitivity of 0%–41% (*195*–*198*). Two studies reported difficulties in visualizing treponemal spirochetes because of background artifacts in silver-stained sections (*196*,*199*).

#### Nucleic Acid Amplification Tests

Although NAATs hold great promise for syphilis diagnosis, especially for primary syphilis, no FDA-cleared NAATs are available for syphilis. Most laboratory-developed NAATs are based on the *tp47* (*tp074*) or *polA* (*tp0105*) genes with varying sensitivities depending on the stage of syphilis and specimen type (*193*,*197*,*202*–*204*). A highly sensitive reverse transcriptase PCR test that targets a region of the 16S rRNA gene has also been described (*205*) and used on CSF in research studies (*206*–*208*). In addition, a real-time, transcription-mediated assay for research use only that targets the 23S rRNA gene (Hologic TMA) has been used to evaluate the presence of *T. pallidum* in rectal and pharyngeal specimens (*108*). Certain laboratories have CLIA-validated PCR tests for *T. pallidum* that can be used to test specimens from genital lesions and CSF. A digital droplet PCR test was recently used to evaluate the presence of *T. pallidum* in saliva (*209*).

The sensitivity of *tp47* and *polA* targets varies across studies, from 72% to 95% on lesion exudate of primary syphilis and from 20% to 86% on secondary lesion swabs depending on lesion type sampled (skin rash versus condylomata lata). These studies are limited by limited sample sizes and different reference standards that include some combination of the following: syphilis clinical diagnosis, serologic findings, or darkfield microscopy results (*109*,*110*,*189*,*203*,*204*,*210*,*211*). If both a darkfield microscopy and a NAAT are performed on the same lesion, the specimen for darkfield microscopy should be collected first. A summary of specimen type and collection, transport, and storage requirements for NAAT specimens drawn from references is presented (Table 2).

A NAAT that targets the *polA* gene had a sensitivity of 84% when tested from maculopapular lesions that were scraped from patients with secondary syphilis using the noncutting edge of a sterilized blade (*112*). The previously described low sensitivity of NAATs in detecting *T. pallidum* from maculopapular lesions might have been attributable to inadequate sampling; however, more studies using this scraping technique for direct detection of *T. pallidum* in skin lesions are required to better estimate NAAT performance. Sensitivities of NAATs on secondary syphilis lesion biopsies vary from 26% to 75%. These studies are limited by different sample collection methods and reference standards, including a combination of clinical, IHC, or serologic findings (*187*,*195*,*197*,*198*); the highest sensitivity was reported using unfixed tissue frozen immediately after collection.

Among 24 MSM, the Hologic TMA demonstrated a sensitivity for rectal and pharyngeal swabs of 41.6% and 29.5% compared with a NAAT targeting *tp47* that was 37.5% and 12.5% sensitive for rectal and pharyngeal swabs, respectively (*108*). Although target sequences for *T. pallidum* NAATs are specific to the organism (*41*) and minimal cross-reactivity with commensal *Treponema* spp. suggests they can be used on oral lesions, more research on target specificity is required to be conclusive. In addition, the *tp47* and *polA* NAATs detect all three pathogenic *T. pallidum* subsp. (*T. pallidum, T. pertenue, and T. endemicum*). A NAAT that distinguishes among these three subspecies has been described but has not been validated with syphilis specimens (*212*).

NAAT sensitivity using whole blood or its components (serum and plasma) or CSF from adults varies considerably and is limited by small sample sizes; additional studies are needed before these sample types can be considered for clinical testing (*110*,*189*,*210*). Compared with RIT, sensitivity of NAATs looks promising for amniotic fluid (75% versus 100%), neonatal CSF (60% versus 75%), and neonatal whole blood or serum (67% versus 94%) in congenital syphilis (*192*,*193*,*213*–*215*). CDC’s *Sexually Transmitted Infections Treatment Guidelines, 2021* suggest that examination of the placenta, umbilical cord, suspicious lesions, nasal discharge, or other body fluids with a CLIA-validated NAAT could be considered in aiding the diagnosis of congenital syphilis (*55*).

NAATs amplifying the *tp47* gene are highly specific (98%–100%) and have been performed on different specimen types, including lesion exudates of primary and secondary syphilis; lesion biopsies of secondary syphilis; CSF from neurosyphilis cases; and whole blood, serum, and plasma from primary, secondary, and latent syphilis cases. Assays targeting the *polA* gene demonstrate similar specificity (98%–100%) and have been performed on lesion exudates of primary and secondary syphilis as well as CSF from neurosyphilis cases (*109*,*110*,*189*,*203*,*204*,*210*,*211*). NAATs with an open platform, regardless of target, are more susceptible than other direct detection tests to false-positive results caused by sample contamination if strict, clean quality control procedures are not used.

On the basis of limited data, laboratory-developed NAATs can be used for primary or possible secondary syphilis lesions (e.g., moist lesions including oral lesions [mucous patches]) in seronegative patients provided that laboratories establish performance specifications to satisfy CMS regulations for CLIA compliance (*109*,*110*,*189*,*204*,*210*,*211*). NAATs might offer more timely diagnosis of primary syphilis compared with serologic testing but have limited additional benefit over serology for secondary syphilis. NAATs can be considered as an adjunct test in amniotic fluid, neonatal CSF, or neonatal blood in cases of suspected congenital infection (*55*,*192*,*193*,*213*–*215*). Although positive NAAT results are helpful in establishing a diagnosis, a negative result in any of these specimens does not rule out infection because of limited sensitivity. NAATs are not recommended for whole blood or blood fractions because of low sensitivity, and data are insufficient to recommend CSF NAAT testing in adults with symptoms suggestive of neurosyphilis (*110*,*189*,*210*). Data are insufficient to recommend their use on ocular fluid or tissue from gummas or other tertiary syphilis lesions.

### Point-of-Care Serologic Testing

Because the syphilis algorithm might require confirmatory or other reflex testing, laboratory-based serologic testing for syphilis might take 3–5 days and might require patients to return to the clinic for follow-up or treatment. An accurate POC serologic antibody test for syphilis can shorten the time to treatment because the infection could be identified at the time of the visit or encounter. Studies evaluating the performance of POC syphilis serologic tests include traditional or reverse algorithms that use nontreponemal (lipoidal antigen) and treponemal laboratory-based serologic tests as reference standards (Supplementary Table 7, https://stacks.cdc.gov/view/cdc/138288). Multiple POC syphilis serologic tests or dual POC serologic tests are available and used internationally for HIV and syphilis (https://www.who.int/publications/i/item/9789240077126); however, only the Syphilis Health Check (Diagnostics Direct) and Dual Path Platform (DPP) HIV-Syphilis Assay (Chembio Diagnostics) are FDA cleared and CLIA waived for the detection of *T. pallidum* antibodies. Physician office laboratories and public health field-based screening programs that offer CLIA-waived tests are required to have and maintain a CLIA certificate of waiver that requires these tests to be quality assured and operated by trained personnel according to manufacturer instructions (https://www.cdc.gov/labquality/waived-tests.html).

#### Syphilis Health Check

In two prospective studies with 202 and 562 participants, the sensitivity and specificity of the Syphilis Health Check ranged from 50.0% to 71.4% and from 91.5% to 95.9%, respectively, when compared with the Trep-Sure EIA as the reference standard (*216*,*217*) (Supplementary Table 7, https://stacks.cdc.gov/view/cdc/138288). When compared with a reference standard of RPR and TPPA in two other studies with 965 and 690 participants conducted in an outreach setting and emergency departments, the Syphilis Health Check had a sensitivity of 76.9% and 90.0% and a specificity of 98.5% and 99.0% (*218*,*219*). In the study with 965 participants, the sensitivity of the Syphilis Health Check was 50.0% and specificity was 99.4% compared with TPPA alone (*219*). The goal of POC testing is to reach populations who might not seek care and might be more likely to have infections that otherwise go undetected and untreated. The results of the two latter studies suggest that the Syphilis Health Check test might be successful in reaching these populations. A 2018 CDC retrospective study used 1,406 archived sera from U.S. commercial and public health laboratories to evaluate the performance of Syphilis Health Check against treponemal tests only (TPPA, EIA, and CIA) and both treponemal and nontreponemal (lipoidal antigen) (RPR) tests in a laboratory setting (*220*). The overall analysis indicated that the sensitivity and specificity of the Syphilis Health Check were 88.7% and 93.1%, respectively, when compared with treponemal tests alone; comparison with both treponemal and nontreponemal (lipoidal antigen) tests demonstrated 95.7% sensitivity and 93.2% specificity. The study demonstrated that the performance of Syphilis Health Check might be comparable to the current treponemal antibody tests used in clinical settings but did not provide performance data on the populations who might have inconsistent health care seeking. In addition, syphilis history and treatment status data were not available for the patients in this retrospective study.

#### DPP HIV-Syphilis Assay

In two studies with 150 and 450 participants that used the FDA-cleared version of the DPP HIV-Syphilis Assay with the DPP Micro Reader, sensitivity and specificity of the DPP HIV-Syphilis Assay for syphilis were 95.3% and 100% and 98.7% and 100%, respectively, when compared with TPPA (*221*,*222*). Although accurate, low-cost rapid tests have the potential to expand testing to populations who otherwise would not be tested in a timely manner, data are insufficient to recommend when and where to use these tests. Further data on the costs and predictive value of POC serologic tests are needed to assess the implementation of tests in settings that serve populations without regular medical care and those with and without a history of treated syphilis. Costs of testing and timely treatment of persons with untreated syphilis in established syphilis screening programs need to be compared with the costs of reaching, testing, and treating populations in outreach settings, emergency departments, or delivery rooms.

### Syphilis Laboratory Test Reporting

#### Reporting to Public Health Departments

Syphilis has important public health implications, and cases are required to be reported to state or local health departments by the health care provider, laboratory, or both, depending on the state public health reporting statutes. Because clinical information might be unavailable to the laboratory, all positive syphilis direct detection tests, along with specimen site and positive syphilis serologic tests, should be reported to state and local health departments. State laws detail which syphilis test results to report and time frames for reporting laboratory results.

Both probable and confirmed cases of syphilis should be reported by health care providers to the local or state health department. Clinical criteria used to stage patients with syphilis might differ from public health surveillance case definitions. Current case definitions are available at https://ndc.services.cdc.gov/case-definitions/syphilis-2018. For surveillance purposes, probable cases are defined as the patient having signs or symptoms consistent with the stage of syphilis and having supportive laboratory test results (e.g., serology) that detect an immune response to the pathogen (*223*). A confirmed case is similar except that the presence of the organism is verified by a direct detection method specific for *T. pallidum*.

#### Reporting to Health Care Providers

When reporting results to health care providers, laboratories should list all tests used, report each result with an interpretation, and document the syphilis algorithm applied to render the interpretation, when appropriate (*224*). Any changes in the test algorithm should be communicated to the submitter and include information about differences in interpretation depending on the test algorithm. Preliminary results released to the submitter should list tests that are pending. All the tests and results should be listed in the final report, even if one or more tests (e.g., the nontreponemal [lipoidal antigen] tests or TPPA) were sent to an outside laboratory.

## Opportunities for Additional Research on the Laboratory Detection of *T. pallidum* Infections

### Serology and CSF Antibody Tests

Serologic antibody tests for syphilis have been the mainstay for syphilis testing in the United States for decades. However, additional research in multiple areas would enhance the utility of current serologic tests.

Studies of test performance are needed to estimate the sensitivity of nontreponemal (lipoidal antigen) tests for primary syphilis against a reference standard of darkfield microscopy or well-characterized NAATs on anogenital lesions. Additional data are needed on serologic test performance in cases of latent syphilis (stratified by duration of infection: early latent, late latent, and latent of unknown duration), late-stage syphilis, symptomatic neurosyphilis, ocular syphilis, and otic syphilis. To conduct these studies, specimen banks of sera that are well characterized by syphilis stage are essential.

Test performance studies of DBS testing compared with laboratory-based treponemal tests would allow assessment of its potential as a diagnostic tool. In addition, establishing cutoff values for signal strength of immunoassays that are likely to be confirmed as true positives for syphilis should be a priority. More studies are needed to determine whether such information would aid in clinical decision-making. Continued research on the performance of the two different serologic testing algorithms in populations with low, medium, and high prevalence of syphilis and the development of a cost-benefit analysis tool would aid in laboratory decision-making when selecting the best approach for their setting. Finally, evaluation of the CSF TPPA in studies with larger sample sizes and in populations with and without syphilis is needed to better assess specificity of the assay. To better determine the test performance characteristics of the CSF antibody tests, head-to-head studies of CSF nontreponemal (lipoidal antigen) and treponemal antibody tests would be conducted with larger samples, using comparable, high-quality, agreed-upon reference standards, and in more populations with well-characterized symptom status.

### Direct Detection Tests

Direct detection of *T. pallidum* has been based on microscopy but is being modernized with molecular methods for detection. No FDA-cleared molecular tests are marketed in the United States, although certain laboratories offer such testing using in-house laboratory-developed and validated tests. Molecular tests that are FDA cleared for *T. pallidum* would facilitate their uptake in laboratories. However, additional research is needed in determining optimal specimen types, including genital and extragenital specimens stratified by stage of syphilis, specimen transport and storage, and specimen adequacy; identifying molecular markers that could be used to monitor for the emergence of antimicrobial resistance and strain typing to better aid epidemiological investigations; evaluating the sensitivity of NAATs on whole blood or its components (serum and plasma); and assessing the cross-reactivity of NAATs with commensal *Treponema* spp.

### POC Tests

Despite years of study internationally, nonlaboratory-based POC tests for syphilis are in their infancy in the United States, with only two FDA-cleared and CLIA-waived tests. Additional POC tests and data are needed to increase understanding of their performance in clinical and outreach settings. Additional areas needed for research include well-designed prospective studies on POC test performance in the context of screening algorithms, special patient populations, linkage to treatment and care, and cost-benefits so that recommendations can be made regarding performance and use in the United States. Also needed are studies comparing POC tests with FDA-cleared laboratory-based treponemal serologic tests, followed by programmatic recommendations for implementation to guide their appropriate use in syphilis testing algorithms.
